# Evaluating yeasts as biocontrol agents against pathogenic and spoilage microorganisms in agri-food: action of volatile organic compounds (VOCs)

**DOI:** 10.1007/s00253-026-13896-w

**Published:** 2026-06-06

**Authors:** Laura Moretti, Laura Canonico, Alice Agarbati, Francesca Comitini, Maurizio Ciani

**Affiliations:** Department of Life and Environmental Sciences (DiSVA), Polytechnic University of Marche, Via Brecce Bianche, 60131 Ancona, Italy

**Keywords:** Yeast, Antibacterial, Anti-mold, Volatile organic compounds

## Abstract

**Abstract:**

Yeasts are widely recognized for their industrial and fermentative relevance, but their potential as source of antibacterial compounds remains significantly underexplored compared to bacteria or filamentous fungi. This study evaluated a wide taxonomically characterized population of yeast strains for their antimicrobial activity against enteropathogenic bacteria, plant pathogens and spoilage molds, using both direct-contact and VOC-mediated assays. Our findings reveal that antimicrobial activity, including the production of inhibitory VOCs, is highly complex, depending not only on the genus and species but also on unique traits at the strain level. *Metschnikowia pulcherrima* exhibited a broad inhibitory spectrum against both molds and bacteria, driven by non-volatile, multifactorial mechanisms such as iron chelation and antimicrobial metabolites. In contrast, *Hanseniaspora* and *Wickerhamomyces* displayed more versatile behaviour, combining strong antifungal properties with VOC-mediated antibacterial activity. *Aureobasidium* and *Meyerozyma* genera showed a functional specialization toward antifungal activity, with limited or no effect on bacterial pathogens. The specific strains of *B. bruxellensis* evaluated in this study emerged as notable producers of bioactive VOCs under the tested in vitro conditions. While these results highlight a significant antagonistic potential, the observed activity appears to be strain-specific. This underscores the importance of intra-specific screening to identify ‘elite’ isolates with superior biocontrol traits, rather than assuming a uniform species-level metabolic profile.

**Key points:**

• *Yeasts antimicrobial activity is genus/species relevant strain-specific traits.*

• *Several yeast genera showed inhibitory activity against enteropathogenic bacteria.*

• *B. bruxellensis represents a promising source of bioactive VOCs against both bacteria and mold.*

**Supplementary Information:**

The online version contains supplementary material available at 10.1007/s00253-026-13896-w.

## Introduction

Current strategies to control foodborne pathogens and spoilage microorganisms rely on a combination of thermal treatments, chemical preservatives, and biological control agents.

Thermal treatments such as pasteurization or sterilization effectively inactivate microorganisms but may adversely affect food quality. Chemical preservatives such as organic acids, nitrites, and sulfites are also widespread, but are now being questioned due to their potential toxicity and consumer preference for additive-free foods. Consequently, bio preservation strategies, including bacteriocins, protective cultures, or natural extracts, have gained significant attention as more sustainable alternatives (Amit et al. 2017; Singh 2018; Elbehiry and Alajaji 2026).

Most antimicrobial molecules currently in use were isolated from soil, particularly *Actinobacteria* (mainly *Streptomyces* species) and *Bacillus*, as well as from filamentous fungi such as *Penicillium* and *Aspergillus* (Elbendary et al. 2018; Omeike et al. 2019; Nuanjohn et al. 2023). These organisms have historically been the most productive sources of antibiotics and bioactive secondary metabolites (Sułkowska-Ziaja et al. 2023). However, the frequent re-isolation of known compounds, the decline in discovery rates, and the rise of multidrug-resistant pathogens have reduced the effectiveness of traditional screening, prompting research into alternative sources such as marine microbes (Srinivasan et al. 2021; Bharathi and Lee 2024), endophytic fungi (Silva et al. 2022), extremophiles (Dishliyska et al. 2025), and other unexplored microbial communities (Quinn and Dyson 2024).

In this context, yeasts have recently emerged as a promising source of antimicrobial molecules, garnering attention for their metabolic versatility and their capacity to synthesize peptides, enzymes, and secondary metabolites with inhibitory potential against foodborne pathogens (Goerges et al. 2006; Hatoum et al. 2012; Ma et al. 2023; Georgescu et al. 2024). Yeasts may exert antimicrobial effects through various mechanisms (Wu et al. 2025): they may compete with pathogens for nutrients and space, secrete extracellular lytic enzymes, and release volatile organic compounds (VOCs). Furthermore, some yeasts also demonstrate resilience to oxidative stress, exhibiting protective biofilm formation or stimulating plant defense mechanisms by inducing the synthesis of phytoalexins and pathogenesis-related proteins (Oztekin et al. 2023; Olana et al. 2025). Yeasts are well known for their ‘killer’ activity, the ability of certain strains to secrete proteinaceous toxins, known as killer toxins or mycocins, capable of inhibiting or killing sensitive yeasts and fungi while leaving the producer strain unaffected (Mannazzu et al. 2019). Beyond general competition, specialized mechanisms have been characterized, such as the iron-chelating activity of pulcherrimin produced by *M. pulcherrima* (Oro et al. 2014; Parafati et al. 2015; Sipiczki 2020) and the secretion of lytic enzymes including β−1,3-glucanase, protease, and chitinase, which degrade the cell walls of sensitive fungi and bacteria (Bar-Shimon et al. 2004). While the application of yeasts as biocontrol agents has been extensively investigated against food spoilage molds and yeasts (Hammami et al. 2022; Esteves et al. 2023), their inhibitory activity toward pathogenic bacteria remains relatively unexplored (Younis et al. 2017; Georgescu et al. 2024). Despite this potential, a systematic screening of yeasts as a primary source of novel antimicrobial compounds is still largely missing. This gap in knowledge is primarily due to the historical focus on specialized metabolites from filamentous fungi and *Actinobacteria*, which has led to a lack of standardized protocols tailored to detect the unique, often contact-independent or VOC-mediated, inhibitory mechanisms of yeasts. Furthermore, the metabolic diversity of yeasts across different food-related genera remains under-characterized, leaving a vast repertoire of strain-specific antimicrobial traits untapped. Considering these factors, we hypothesized that yeasts from diverse and competitive food environments exhibit genus- and strain-specific antimicrobial properties. Specifically, we investigated their ability to produce inhibitory VOCs to control spoilage and pathogenic microbiota.

## Materials and methods

### Microorganisms and culture conditions

A total of 107 yeasts from the Yeast Collection of the Department of Life and Environmental Sciences (DiSVA) of the Polytechnic University of Marche (Italy) were used. The yeast strains investigated in this study were previously isolated from various food matrices and environmental samples.

Whole information regarding strain codes, species identification, and isolation sources is provided in Table 1. Stock cultures were preserved at −80 °C in YPD broth (5 g/L yeast extract, 10 g/L peptone, and 20 g/L glucose) supplemented with 20% (v/v) glycerol. For experimental use, isolates were recovered on YPD agar, incubated at 25 °C, and subsequently maintained at 4 °C for short-term storage.

**Table 1:** DiSVA code, species, and source of isolation of strains used to evaluate the antimicrobial activity against pathogen and spoilage, bacteria, and fungi

DiSVA code	Species	Source
DiSVA 211	*Aureobasidium pullulans*	Plant surface
DiSVA 810	*Brettanomyces bruxellensis*	Gueze beer
DiSVA 812	*Brettanomyces bruxellensis*	Gueze beer
DiSVA 814	*Brettanomyces bruxellensis*	Gueze beer
DiSVA 816	*Brettanomyces bruxellensis*	Gueze beer
DiSVA 673	*Candida homilentoma*	Fossa cheese
DiSVA 670	*Candida zeylanoides*	Fossa cheese
DiSVA 1142	*Candida zeylanoides*	Pecorino cheese
DiSVA 672	*Debaryomyces hansenii*	Fossa cheese
DiSVA 1172	*Debaryomyces hansenii*	Pecorino cheese
DiSVA 1199	*Debaryomyces hansenii*	Artisan dairy
DiSVA 1175	*Debaryomyces hansenii*	Pecorino cheese
DiSVA 1198	*Debaryomyces hansenii*	Pecorino cheese
DiSVA 1165	*Debaryomyces hansenii*	Artisan dairy
DiSVA 604	*Filobasidium magnum*	Fig fruit
DiSVA 801	*Hanseniaspora osmophila*	Winery
DiSVA 800	*Hanseniaspora* spp.	Winery
DiSVA 605	*Hanseniaspora uvarum*	Fig fruit
DiSVA 793	*Kazachstania unispora*	Sourdough homemade
DiSVA 794	*Kazachstania unispora*	Sourdough homemade
DiSVA 795	*Kazachstania unispora*	Sourdough homemade
DiSVA 796	*Kazachstania unispora*	Sourdough homemade
DiSVA 797	*Kazachstania unispora*	Sourdough homemade
DiSVA 798	*Kazachstania unispora*	Sourdough homemade
DiSVA 799	*Kazachstania unispora*	Sourdough homemade
DiSVA 632	*Kluyveromyces marxianus*	Barley Malt
DiSVA 634	*Kluyveromyces marxianus*	Barley Malt
DiSVA 322	*Lachancea thermotolerans*	Grapes
DiSVA 323	*Lachancea thermotolerans*	Grapes
DiSVA 324	*Lachancea thermotolerans*	Grapes
DiSVA 5	*Lachancea thermotolerans*	Grapes
DiSVA 325	*Lachancea thermotolerans*	Grapes
DiSVA 326	*Lachancea thermotolerans*	Wine
DiSVA 723	*Lachancea thermotolerans*	Oak moss
DiSVA 725	*Lachancea thermotolerans*	Oak moss
DiSVA 718	*Lachancea waltii*	Oak moss
DiSVA 733	*Metschnikowia aff. fructicola*	Beech tree bark
DiSVA 738	*Metschnikowia pulcherrima*	Beech tree bark
DiSVA 742	*Metschnikowia pulcherrima*	Farnia bark
DiSVA 606	*Metschnikowia pulcherrima*	Blackthorns
DiSVA 269	*Metschnikowia pulcherrima*	Raisin berry surface
DiSVA 267	*Metschnikowia pulcherrima*	Grape berry surface
DiSVA 746	*Metschnikowia reukaufii*	Farnia bark
DiSVA 732	*Metschnikowia ziziphicola*	Beech tree bark
DiSVA 1283	*Meyerozyma caribbica*	Honey bees
DiSVA 1284	*Meyerozyma caribbica*	Honey bees
DiSVA1341	*Meyerozyma caribbica*	Honey bees
DiSVA 1285	*Meyerozyma caribbica*	Honey bees
DiSVA 1288	*Meyerozyma guilliermondii*	Honey bees
DiSVA 1295	*Meyerozyma guilliermondii*	Honey bees
DiSVA 1296	*Meyerozyma guilliermondii*	Honey bees
DiSVA 671	*Pichia anomala*	Fossa cheese
DiSVA 628	*Pichia fermentans*	Malt
DiSVA 630	*Pichia fermentans*	Malt
DiSVA 609	*Pichia manshurica*	Grapes
DiSVA 769	*Saccharomyces bayanus*	Verdicchio wine
DiSVA 770	*Saccharomyces bayanus*	Verdicchio wine
DiSVA 674	*Saccharomyces cerevisiae*	Fossa cheese
DiSVA 716	*Saccharomyces cerevisiae*	Oak moss
DiSVA 788	*Saccharomyces cerevisiae*	Sourdough homemade
DiSVA 860	*Saccharomyces cerevisiae*	Sourdough homemade
DiSVA 861	*Saccharomyces cerevisiae*	Sourdough homemade
DiSVA 862	*Saccharomyces cerevisiae*	Sourdough homemade
DiSVA 863	*Saccharomyces cerevisiae*	Sourdough homemade
DiSVA 864	*Saccharomyces cerevisiae*	Sourdough homemade
DiSVA 789	*Saccharomyces cerevisiae*	Sourdough homemade
DiSVA 790	*Saccharomyces cerevisiae*	Sourdough homemade
DiSVA 865	*Saccharomyces cerevisiae*	Sourdough homemade
DiSVA 791	*Saccharomyces cerevisiae*	Sourdough homemade
DiSVA 792	*Saccharomyces cerevisiae*	Sourdough homemade
DiSVA 866	*Saccharomyces cerevisiae*	Sourdough homemade
DiSVA 772	*Saccharomyces cerevisiae*	Verdicchio wine
DiSVA 780	*Saccharomyces cerevisiae*	Verdicchio wine
DiSVA 774	*Saccharomyces cerevisiae*	Verdicchio wine
DiSVA 767	*Saccharomyces cerevisiae*	Verdicchio wine
DiSVA 778	*Saccharomyces cerevisiae*	Verdicchio wine
DiSVA 766	*Saccharomyces cerevisiae*	Verdicchio wine
DiSVA 764	*Saccharomyces cerevisiae*	Verdicchio wine
DiSVA 777	*Saccharomyces cerevisiae*	Verdicchio wine
DiSVA 763	*Saccharomyces cerevisiae*	Verdicchio wine
DiSVA 779	*Saccharomyces cerevisiae*	Verdicchio wine
DiSVA 776	*Saccharomyces cerevisiae*	Verdicchio wine
DiSVA 719	*Scheffersomyces shehatae*	Oak moss
DiSVA 720	*Scheffersomyces shehatae*	Oak moss
DiSVA 734	*Scheffersomyces shehatae*	Oak moss
DiSVA 717	*Torulaspora delbrueckii*	Oak moss
DiSVA 426	*Torulaspora delbrueckii*	Sugar cane juice
DiSVA 399	*Torulaspora delbrueckii*	Corrosol fruit
DiSVA 413	*Torulaspora delbrueckii*	Papaya leaves
DiSVA 602	*Torulaspora delbrueckii*	Papaya leaves
DiSVA 419	*Torulaspora delbrueckii*	Papaya leaves
DiSVA 445	*Torulaspora delbrueckii*	Coconut palm
DiSVA 432	*Torulaspora delbrueckii*	Sugar cane juice
DiSVA 363	*Torulaspora delbrueckii*	Sugar cane juice
DiSVA 254	*Torulaspora delbrueckii*	Papaya leaves
DiSVA 255	*Torulaspora delbrueckii*	Corrosol fruit
DiSVA 256	*Torulaspora delbrueckii*	Sugar cane juice
DiSVA 258	*Torulaspora delbrueckii*	Grapes
DiSVA 260	*Torulaspora delbrueckii*	Grapes
DiSVA 261	*Torulaspora delbrueckii*	Grapes
DiSVA 313	*Torulaspora delbrueckii*	Grapes
DiSVA 315	*Torulaspora delbrueckii*	Winery surface
DiSVA 55	*Torulaspora delbrueckii*	Soil
DiSVA 343	*Torulaspora delbrueckii*	Papaya leaves
DiSVA 130	*Torulaspora delbrueckii*	Winery surface
DiSVA 671	*Wickerhamomyces anomalus*	Fossa cheese
DiSVA 2	*Wickerhamomyces anomalus*	Baker’s yeast

### In vitro antimicrobial assays

#### Killer yeast test assay

Killer activity of yeast strains was evaluated using a plate assay with *S. cerevisiae* DiSVA 42 (NCYC 1006, National Collection of Yeast Cultures) and *B. bruxellensis* DiSVA 46 as sensitive strains.

The assay medium (Malt Agar, adjusted to pH 4.2) was inoculated with the sensitive strains at a final concentration of 10^6^ CFU/mL. Test yeast strains were spotted as fresh cultures; after 48 h of incubation at 25 °C, antimicrobial activity was assessed based on the appearance of distinct inhibition zones.

Results were recorded as the presence (+) or absence (−) of an inhibition zone, according to the method described by (Comitini et al. 2020).

#### Antibacterial in vitro assay

The antimicrobial activity of the isolates was assessed against four target pathogens: *Escherichia coli*, *Listeria monocytogenes*, *Salmonella enterica*, and *Staphylococcus aureus*, all sourced from the DiSVA Microbial Collection of the Polytechnic University of Marche.

Bacterial cultures were pre-cultured in Plate Count Broth (tryptone 5 g/L, yeast extract 2.5 g/L, glucose 1 g/L) at 37 °C for 24 h.

Standardized yeasts suspension (70 µL) corresponding to 10^7^ CFU/mL s were inoculated into wells, previously formed on YPD agar and incubated at 30 °C for 48 h. Subsequently, a soft layer of nutrient agar (10 mL; beef extract 3 g/L, peptone 5 g/L, agar 15 g/L) previously inoculated with 1 mL of each pathogen culture at concentration of 10^5^ CFU/mL was then poured over the surface, forming a bilayer. After incubation at 37 °C for 24 h, antimicrobial activity was identified by the presence of clear inhibition zones. *S. boulardii* (CODEX, Zambon) served as a positive control, while plates without yeast inoculation were used as negative controls. The results were expressed by evaluating the diameter of inhibition halos around each well (Agarbati et al. 2020). The assay was conducted in triplicate.

#### Anti-mold in vitro assay

The anti-mold activity of yeast strains was evaluated following the method described by Tryfinopoulou et al. (2020), with slight modifications. Briefly, fresh yeast cultures were streaked on Potato Dextrose Agar (PDA) plates (90 mm) forming a circular line of approximately 60 mm in diameter and incubated at 25 °C for 48 h. Subsequently, 10-µL spots of *Botrytis cinerea*, *Aspergillus carbonarius*, *Penicillium expansum*, *Penicillium digitatum*, and *Cladosporium* spp. (DiSVA Microbial Collection of the Polytechnic University of Marche) spore suspension (10^6^ spores/mL in 0.1% Triton X-100) were inoculated at the centre of the yeast ring. Plates were incubated at 25 °C for 7 days. *A. pullulans* strains (Botector, Manica Spa) served as a positive control, while a plate without yeast inoculation was used as negative control. Results were evaluated by measuring the radial growth of the mold and expressed as the percentage of mycelial growth inhibition, calculated according to the following formula:

$$I\mathrm{\%}=\frac{{R}_{0}-{R}_{x}}{{R}_{0}}\times 100$$

where *R*_0_ represents the radial growth of mold in negative control, and *Rₓ* the growth in the presence of the yeast. The assay was conducted in triplicate.

### Antimicrobial activity mediated by volatile organic compounds (VOCs)

To assess antibacterial activity mediated by VOCs, the method proposed by Parafati et al. (2015) was adopted with appropriate modifications to enable the use of bacteria instead of molds. A yeast colony in the exponential growth phase was spread on a YPD plate to achieve confluent growth. The plate was coupled with a clean PCA plate, placing them face to face, sealed with parafilm, and incubated at 25 °C for 48 h. Subsequently, a 10 µL of a standardized bacterial suspension (10^5^ CFU/mL) of *E. coli*, *L. monocytogenes*, *S. enterica*, and *S. aureus* was spotted on the surface of the PCA plate and immediately sealed again with parafilm. The assembly was incubated at 37 °C for 24 h. *S. boulardii* (CODEX, Zambon) served as a positive control, while sterile distilled water was used in place of yeast as a negative control. Results were recorded based on the presence (+), absence (−) or partial inhibition (±) of bacterial growth at the inoculation sites. The assay was conducted in triplicate.

### Antifungal activity mediated by  VOCs

To evaluate the effect of yeast volatile compounds on mold growth, a yeast colony in the exponential growth phase was spread on a YPD plate to obtain confluent growth and incubated at 25 °C for 48 h. Subsequently, a 10 µL spot of *B. cinerea*, *A. carbonarius*, *P. expansum*, *P. digitatum*, and *Cladosporium* spore suspensions (10^6^ spores/mL in 0.1% Triton X-100) was inoculated at the centre of PDA plates and was left to dry. Finally, the lids of both Petri dishes were removed, and plates were placed facing each other and sealed with Parafilm to allow interaction through volatile compounds. The plates were incubated at 25 °C for 7 days. *A. pullulans* (Botector, Manica Spa) served as a positive control, while a plate without yeast inoculation was used as negative control. Results were expressed as the percentage inhibition (I%) of mold radial growth, calculated as previously described in paragraph 2.2.3. (Acar et al. 2024; Parafati et al. 2015). The assay was conducted in triplicate.

### Statistical analysis

Graphical visualizations (boxplot), Mann-Whitney *U*-test, and Duncan’s test were performed using R (R Core Team, Vienna, Austria).

Principal component analysis (PCA) was applied to discriminate the activity of VOCs by yeast strains against bacteria and fungi. The statistical software Chemometric Agile Tool (CAT, Leardi R et al.) was used for statistical analysis. The data were centred and scaled (z-score normalization), ensuring that all variables contributed equally to the analysis regardless of their original measurement scale.

## Results

### Killer and antibacterial activity assays

The antibacterial activity and killer activity of 107 yeast strains belonging to 16 genera and 26 species are reported in Table 2.

**Table 2:** Antibacterial activity against *E. coli*, *S. aureus*, *S. enteritidis*, *L. monocytogenes*, and killer activity of yeast strains against the sensitive strains *B. bruxellensis* DiSVA 46 and *S. cerevisiae* DiSVA 42. Results are reported as presence (+) or absence (−) of sensitive yeast strains growth for killer activity. Antimicrobial activity is expressed as inhibition halo diameter (mm), values are means ± standard deviations

DiSVA code	Species	*E. coli*	Antibacterial activity			Killer activity	
			*S. aureus*	*S. enteritidis*	*L. monocytogenes*	*B. bruxellensis*	*S. cerevisiae*
DiSVA 211	*A. pullulans*	0 ± 0	0 ± 0	0 ± 0	0 ± 0	−	−
DiSVA 810	*B. bruxellensis*	29 ± 2	27 ± 2	42 ± 8	28 ± 2	−	−
DiSVA 812	*B. bruxellensis*	33 ± 2	31 ± 0	40 ± 2	24 ± 0	−	−
DiSVA 814	*B. bruxellensis*	27 ± 2	28 ± 4.5	34 ± 0	18 ± 0	−	−
DiSVA 816	*B. bruxellensis*	27 ± 2	17 ± 0.5	33 ± 2	14 ± 2	−	−
DiSVA 673	*C. homilentoma*	29 ± 0	0 ± 0	15 ± 2	24 ± 2	−	+
DiSVA 670	*C. zeylanoides*	0 ± 0	0 ± 0	0 ± 0	0 ± 0	−	−
DiSVA 1142	*C. zeylanoides*	0 ± 0	0 ± 0	0 ± 0	0 ± 0	−	−
DiSVA 672	*D. hansenii*	0 ± 0	0 ± 0	0 ± 0	0 ± 0	−	+
DiSVA 1172	*D. hansenii*	0 ± 0	0 ± 0	0 ± 0	0 ± 0	−	+
DiSVA 1199	*D. hansenii*	0 ± 0	0 ± 0	17 ± 2	0 ± 0	−	−
DiSVA 1175	*D. hansenii*	0 ± 0	0 ± 0	0 ± 0	0 ± 0	−	−
DiSVA 1198	*D. hansenii*	0 ± 0	0 ± 0	0 ± 0	0 ± 0	−	+
DiSVA 1165	*D. hansenii*	0 ± 0	0 ± 0	0 ± 0	0 ± 0	−	−
DiSVA 604	*F. magnum*	0 ± 0	0 ± 0	0 ± 0	25 ± 2	−	−
DiSVA 801	*H. osmophila*	0 ± 0	16 ± 0.5	24 ± 2	13 ± 2	−	−
DiSVA 800	*Hanseniaspora* spp.	18 ± 0	19 ± 4.5	22 ± 2	15 ± 0	−	−
DiSVA 605	*H. uvarum*	24 ± 2	14 ± 0	24 ± 2	0 ± 0	−	−
DiSVA 793	*K. unispora*	0 ± 0	0 ± 0	0 ± 0	0 ± 0	−	−
DiSVA 794	*K. unispora*	0 ± 0	0 ± 0	0 ± 0	0 ± 0	−	−
DiSVA 795	*K. unispora*	0 ± 0	0 ± 0	0 ± 0	0 ± 0	−	−
DiSVA 796	*K. unispora*	0 ± 0	0 ± 0	0 ± 0	0 ± 0	−	−
DiSVA 797	*K. unispora*	0 ± 0	0 ± 0	0 ± 0	0 ± 0	−	−
DiSVA 798	*K. unispora*	0 ± 0	0 ± 0	0 ± 0	0 ± 0	−	−
DiSVA 799	*K. unispora*	0 ± 0	0 ± 0	0 ± 0	0 ± 0	−	−
DiSVA 632	*K. marxianus*	0 ± 0	0 ± 0	0 ± 0	0 ± 0	−	−
DiSVA 634	*K. marxianus*	0 ± 0	0 ± 0	20 ± 0	0 ± 0	−	−
DiSVA 322	*L. thermotolerans*	0 ± 0	0 ± 0	0 ± 0	0 ± 0	−	−
DiSVA 323	*L. thermotolerans*	0 ± 0	0 ± 0	0 ± 0	0 ± 0	−	−
DiSVA 324	*L. thermotolerans*	0 ± 0	0 ± 0	0 ± 0	0 ± 0	−	−
DiSVA 5	*L. thermotolerans*	25 ± 0	0 ± 0	0 ± 0	36 ± 2	−	−
DiSVA 325	*L. thermotolerans*	15 ± 0	0 ± 0	0 ± 0	0 ± 0	−	−
DiSVA 326	*L. thermotolerans*	0 ± 0	0 ± 0	0 ± 0	39 ± 2	−	−
DiSVA 723	*L. thermotolerans*	0 ± 0	0 ± 0	0 ± 0	0 ± 0	−	−
DiSVA 725	*L. thermotolerans*	0 ± 0	0 ± 0	0 ± 0	0 ± 0	−	−
DiSVA 718	*L. waltii*	0 ± 0	0 ± 0	0 ± 0	0 ± 0	−	−
DiSVA 733	*M. aff. fructicola*	41 ± 2	14 ± 0.5	36 ± 2	0 ± 0	−	−
DiSVA 738	*M. pulcherrima*	31 ± 2	0 ± 0	24 ± 0	0 ± 0	−	−
DiSVA 742	*M. pulcherrima*	0 ± 0	15 ± 2	0 ± 0	0 ± 0	−	−
DiSVA 606	*M. pulcherrima*	12 ± 0	21 ± 0,5	13 ± 0	0 ± 0	+	−
DiSVA 269	*M. pulcherrima*	0 ± 0	0 ± 0	0 ± 0	9 ± 2	+	+
DiSVA 267	*M. pulcherrima*	0 ± 0	0 ± 0	0 ± 0	0 ± 0	−	−
DiSVA 746	*M. reukaufii*	0 ± 0	0 ± 0	0 ± 0	33 ± 0	−	−
DiSVA 732	*M. ziziphicola*	38 ± 2	0 ± 0	23 ± 2	0 ± 0	−	−
DiSVA 1283	*M. caribbica*	0 ± 0	0 ± 0	0 ± 0	0 ± 0	−	−
DiSVA 1284	*M. caribbica*	0 ± 0	0 ± 0	0 ± 0	0 ± 0	−	−
DiSVA 1341	*M. caribbica*	0 ± 0	0 ± 0	10 ± 2	0 ± 0	−	−
DiSVA 1285	*M. caribbica*	0 ± 0	0 ± 0	9 ± 0	0 ± 0	−	−
DiSVA 1288	*M. guilliermondii*	0 ± 0	0 ± 0	0 ± 0	0 ± 0	−	−
DiSVA 1295	*M. guilliermondii*	0 ± 0	0 ± 0	0 ± 0	0 ± 0	−	−
DiSVA 1296	*M. guilliermondii*	0 ± 0	0 ± 0	0 ± 0	0 ± 0	−	−
DiSVA 671	*P. anomala*	12 ± 0	0 ± 0	16 ± 2	0 ± 0	−	+
DiSVA 628	*P. fermentans*	16 ± 2	0 ± 0	21 ± 2	0 ± 0	−	+
DiSVA 630	*P. fermentans*	20 ± 2	0 ± 0	20 ± 0	23 ± 2	−	+
DiSVA 609	*P. manshurica*	20 ± 2	12 ± 0	13 ± 2	29 ± 0	−	+
DiSVA 769	*S. bayanus*	0 ± 0	0 ± 0	0 ± 0	0 ± 0	−	+
DiSVA 770	*S. bayanus*	14 ± 0	0 ± 0	15 ± 2	0 ± 0	−	+
DiSVA 674	*S. cerevisiae*	0 ± 0	0 ± 0	0 ± 0	0 ± 0	−	+
DiSVA 716	*S. cerevisiae*	0 ± 0	0 ± 0	0 ± 0	0 ± 0	−	−
DiSVA 788	*S. cerevisiae*	0 ± 0	0 ± 0	0 ± 0	0 ± 0	−	−
DiSVA 860	*S. cerevisiae*	0 ± 0	0 ± 0	0 ± 0	0 ± 0	−	−
DiSVA 861	*S. cerevisiae*	0 ± 0	0 ± 0	0 ± 0	0 ± 0	−	−
DiSVA 862	*S. cerevisiae*	0 ± 0	0 ± 0	0 ± 0	0 ± 0	−	−
DiSVA 863	*S. cerevisiae*	0 ± 0	0 ± 0	0 ± 0	0 ± 0	−	−
DiSVA 864	*S. cerevisiae*	0 ± 0	0 ± 0	0 ± 0	0 ± 0	−	−
DiSVA 789	*S. cerevisiae*	0 ± 0	0 ± 0	0 ± 0	0 ± 0	−	−
DiSVA 790	*S. cerevisiae*	0 ± 0	0 ± 0	0 ± 0	0 ± 0	−	−
DiSVA 865	*S. cerevisiae*	0 ± 0	0 ± 0	0 ± 0	0 ± 0	−	−
DiSVA 791	*S. cerevisiae*	0 ± 0	0 ± 0	0 ± 0	0 ± 0	−	−
DiSVA 792	*S. cerevisiae*	0 ± 0	0 ± 0	0 ± 0	0 ± 0	−	−
DiSVA 866	*S. cerevisiae*	0 ± 0	0 ± 0	0 ± 0	0 ± 0	−	+
DiSVA 772	*S. cerevisiae*	17 ± 0	0 ± 0	0 ± 0	0 ± 0	−	−
DiSVA 780	*S. cerevisiae*	10 ± 0	0 ± 0	0 ± 0	0 ± 0	−	+
DiSVA 774	*S. cerevisiae*	16 ± 2	0 ± 0	0 ± 0	0 ± 0	−	+
DiSVA 767	*S. cerevisiae*	0 ± 0	0 ± 0	0 ± 0	0 ± 0	−	+
DiSVA 778	*S. cerevisiae*	0 ± 0	0 ± 0	0 ± 0	0 ± 0	−	+
DiSVA 766	*S. cerevisiae*	0 ± 0	0 ± 0	0 ± 0	0 ± 0	−	+
DiSVA 764	*S. cerevisiae*	0 ± 0	0 ± 0	0 ± 0	0 ± 0	−	+
DiSVA 777	*S. cerevisiae*	0 ± 0	0 ± 0	0 ± 0	0 ± 0	−	+
DiSVA 763	*S. cerevisiae*	0 ± 0	0 ± 0	0 ± 0	0 ± 0	−	−
DiSVA 779	*S. cerevisiae*	0 ± 0	0 ± 0	0 ± 0	0 ± 0	−	+
DiSVA 776	*S. cerevisiae*	0 ± 0	0 ± 0	0 ± 0	0 ± 0	−	−
DiSVA 719	*S. shehatae*	0 ± 0	0 ± 0	0 ± 0	0 ± 0	−	−
DiSVA 720	*S. shehatae*	0 ± 0	0 ± 0	0 ± 0	0 ± 0	−	−
DiSVA 734	*S. shehatae*	0 ± 0	0 ± 0	0 ± 0	0 ± 0	−	−
DiSVA 717	*T. delbrueckii*	13 ± 2	0 ± 0	11 ± 0	0 ± 0	−	−
DiSVA 426	*T. delbrueckii*	11 ± 2	17 ± 2	13 ± 2	0 ± 0	−	−
DiSVA 399	*T. delbrueckii*	13 ± 0	15 ± 2	14 ± 8	0 ± 0	−	−
DiSVA 413	*T. delbrueckii*	15 ± 2	0 ± 0	17 ± 2	0 ± 0	−	−
DiSVA 602	*T. delbrueckii*	0 ± 0	0 ± 0	12 ± 2	0 ± 0	−	−
DiSVA 419	*T. delbrueckii*	15 ± 2	0 ± 0	0 ± 0	0 ± 0	−	−
DiSVA 445	*T. delbrueckii*	0 ± 0	0 ± 0	15 ± 2	0 ± 0	−	−
DiSVA 432	*T. delbrueckii*	13 ± 2	0 ± 0	15 ± 2	0 ± 0	−	−
DiSVA 363	*T. delbrueckii*	13 ± 0	0 ± 0	14 ± 0	0 ± 0	−	−
DiSVA 254	*T. delbrueckii*	0 ± 0	0 ± 0	0 ± 0	0 ± 0	−	−
DiSVA 255	*T. delbrueckii*	12 ± 2	0 ± 0	13 ± 0	0 ± 0	−	−
DiSVA 256	*T. delbrueckii*	15 ± 0	0 ± 0	0 ± 0	0 ± 0	−	−
DiSVA 258	*T. delbrueckii*	18 ± 2	0 ± 0	14 ± 2	0 ± 0	−	−
DiSVA 260	*T. delbrueckii*	0 ± 0	22 ± 4.5	15 ± 2	0 ± 0	−	−
DiSVA 261	*T. delbrueckii*	13 ± 2	0 ± 0	12 ± 2	0 ± 0	−	−
DiSVA 313	*T. delbrueckii*	12 ± 2	0 ± 0	0 ± 0	0 ± 0	−	−
DiSVA 315	*T. delbrueckii*	10 ± 2	0 ± 0	0 ± 0	0 ± 0	−	−
DiSVA 55	*T. delbrueckii*	14 ± 0	0 ± 0	0 ± 0	0 ± 0	−	−
DiSVA 343	*T. delbrueckii*	15 ± 2	0 ± 0	13 ± 2	0 ± 0	−	−
DiSVA 130	*T. delbrueckii*	0 ± 0	0 ± 0	0 ± 0	0 ± 0	−	−
DiSVA 671	*W. anomalus*	27 ± 2	0 ± 0	26 ± 2	0 ± 0	−	−
DiSVA 2	*W. anomalus*	24 ± 2	15 ± 0,5	27 ± 2	0 ± 0	−	−
Control	*A. pullulans*	19 ± 0	9 ± 2	18 ± 2	0 ± 0	−	−
Control	*S. boulardii*	15 ± 2	0 ± 0	13 ± 2	0 ± 0	−	−

The overall results of killer assay showed that *M. pulcherrima* strain DiSVA 606 an DiSVA 269 exhibited killer action against the sensitive type strain of *B. bruxellensis* whereas the sensitive strain of *S. cerevisiae* showed a broader susceptibility spectrum toward different yeast genera. Indeed, the genera *Pichia*, *Debaryomyces*, *Candida*, *Torulaspora*, and *Saccharomyces* (12 strains) exhibited an inhibitory action toward *S. cerevisiae* sensitive test strain (DiSVA 42).

The antibacterial activity of the yeast isolates showed marked variability among species and strains (Table 2). Inhibition was most frequently observed against the Gram-negative bacteria *E. coli* and *S. enteritidis*, which were inhibited by 43.9% and 37.4% of the tested yeast strains, respectively. In contrast, inhibition of the Gram-positive bacteria *S. aureus* and *L. monocytogenes* was less frequently observed, occurring in 18.7% and 16.8% of the tested strains, respectively.

**Fig. 1:**
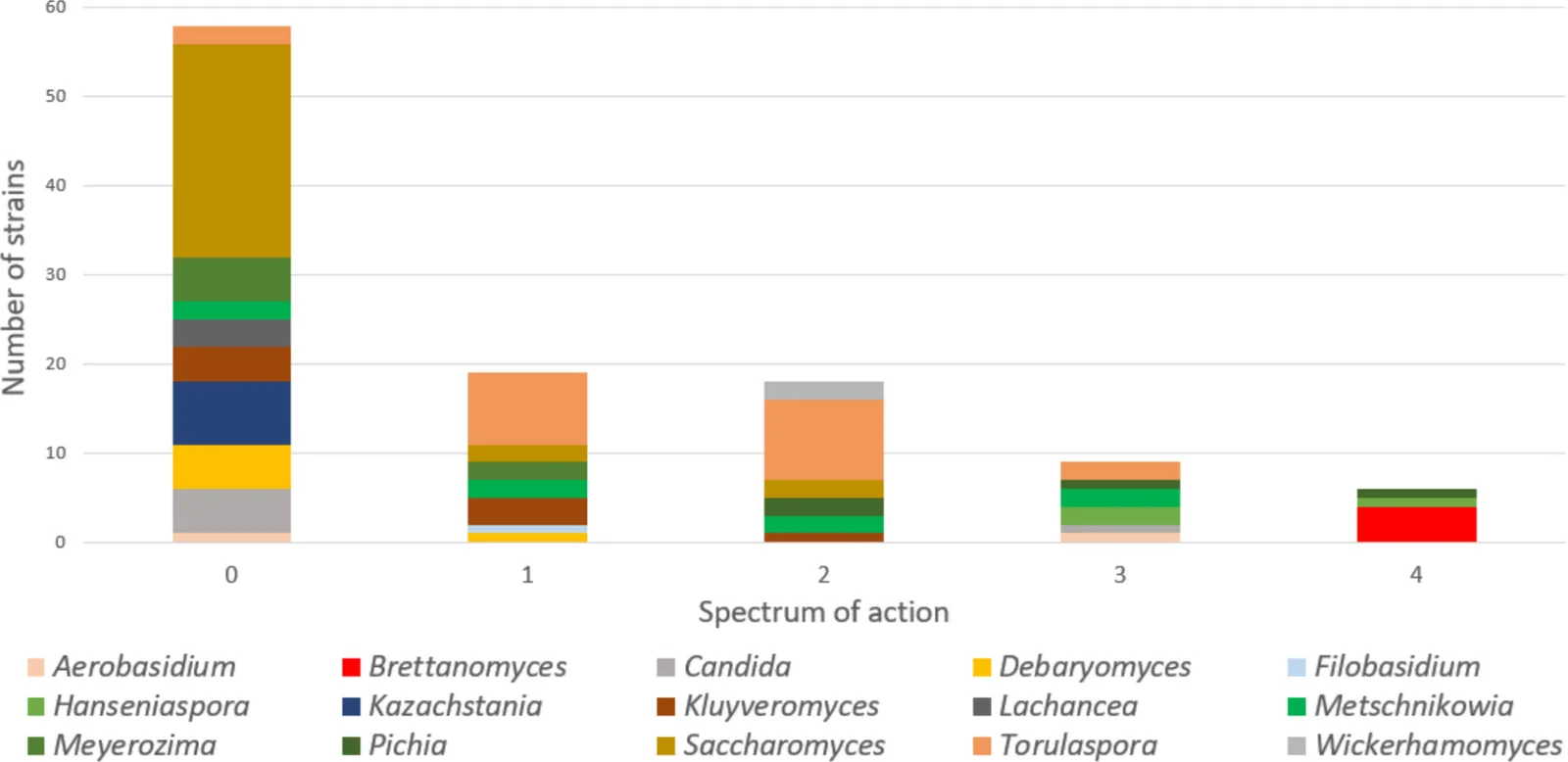
Yeast strains are grouped by genera and distributed according to the number of enteropathogenic bacteria inhibited (*E. coli*, *S. enteritidis*, *S. aureus*, and *L. monocytogenes*). The *x*-axis represents the number of pathogens inhibited, from 0 to 4 (0 = no action; 4 = action against all 4 bacteria), while the *y*-axis indicates the number of strains belonging to each genus

An elaboration of the antibacterial activity results is shown in Fig. 1, where yeast strains are grouped by genus according to the number of enteropathogenic bacteria inhibited. Most yeast genera exhibited low or no antimicrobial activity: *Saccharomyces* (24 out of 28 strains), *Kazachstania* (7 strains), *Debaryomyces* (5 strains), *Candida* (5 strains), *Meyerozyma* (5 strains), and *Lachancea* (3 strains) did not show inhibition. This trend suggested that the absence of antimicrobial activity is a common trait within these genera. Otherwise, other genera displayed marked strain-specific antibacterial activity: while strains belonging to genera *Kluyveromyces* showed a limited inhibition spectrum, all strains belonging to *Torulaspora* (21 strains) and *Metschnikowia* (8 strains) exhibited a wide range of antibacterial activity. This group included strains with no detectable activity as well as strains exhibiting a broader inhibition spectrum, affecting up to 3 or 4 bacterial pathogens. These results indicate that, within these genera, antimicrobial activity is strongly strain-dependent.

The genera *Hanseniaspora* and *Pichia* showed a more widespread inhibitory action, with some strains able to inhibit all the four pathogens tested.

The antimicrobial activity displayed by the *Brettanomyces* strains against enteropathogenic bacteria suggests an interesting inhibitory potential; however, further studies are needed to determine if this trait is consistently maintained across the genus.

### Anti-mold in vitro assay

In Table 3, the percentage reduction of mold growth observed against five tested mold species is reported.

**Table 3:** Growth reduction (%) of mycelial radial growth of *P. expansum*, *B. cinerea*, *P. digitatum*, *Cladosporium* spp., and *A. carbonarius* against all tested yeast strains. Values are means ± standard deviations

DiSVA code	Species	Growth reduction (%)				
		*P. expansum*	*B. cinerea*	*P. digitatum*	*Cladosporium* spp.	*A. carbonarius*
DiSVA 211	*A. pullulans*	34.0 ± 2	73.0 ± 0	52.0 ± 2	43.5 ± 4.5	69.0 ± 0
DiSVA 810	*B. bruxellensis*	47.0 ± 2	99.5 ± 0.5	85.0 ± 0	43.5 ± 4.5	44.5 ± 4.5
DiSVA 812	*B. bruxellensis*	46.0 ± 2	77.5 ± 4.5	93.0 ± 8	73.0 ± 2	55.0 ± 0
DiSVA 814	*B. bruxellensis*	39.0 ± 0	83.0 ± 2	66.5 ± 0.5	62.5 ± 4.5	43.5 ± 0.5
DiSVA 816	*B. bruxellensis*	30.5 ± 0.5	65.0 ± 2	93.5 ± 4.5	77.0 ± 0	43.5 ± 4.5
DiSVA 673	*C. homilentoma*	22.5 ± 4.5	75.5 ± 4.5	28.5 ± 0.5	24.0 ± 8	33.5 ± 4.5
DiSVA 670	*C. zeylanoides*	19.5 ± 4.5	52.0 ± 0	27.5 ± 0.5	21.0 ± 8	12.5 ± 0.5
DiSVA 1142	*C. zeylanoides*	22.5 ± 4.5	49.0 ± 2	32.0 ± 2	21.0 ± 8	25.5 ± 4.5
DiSVA 672	*D. hansenii*	17.5 ± 0.5	68.5 ± 4.5	16.0 ± 8	14.5 ± 4.5	11.0 ± 2
DiSVA 1172	*D. hansenii*	23.5 ± 0.5	45.5 ± 0.5	37.5 ± 0.5	25.5 ± 0.5	19.5 ± 0.5
DiSVA 1199	*D. hansenii*	27.5 ± 0.5	61.0 ± 0	45.5 ± 4.5	17.5 ± 4.5	8.5 ± 4.5
DiSVA 1175	*D. hansenii*	25.5 ± 4.5	77.5 ± 4.5	57.0 ± 8	26.0 ± 0	23.5 ± 4.5
DiSVA 1198	*D. hansenii*	28 ± 2	44 ± 2	29.5 ± 4.5	27.5 ± 4.5	18.5 ± 4.5
DiSVA 1165	*D. hansenii*	27.5 ± 0.5	59.5 ± 4.5	76.0 ± 8	20.5 ± 4.5	19.0 ± 2
DiSVA 717	*F. magnum*	21.5 ± 0.5	44.5 ± 4.5	15.5 ± 0.5	11.5 ± 4.5	17.5 ± 0.5
DiSVA 801	*H. osmophila*	31.5 ± 4.5	69.5 ± 4.5	36.0 ± 0	53.5 ± 4.5	20.5 ± 4.5
DiSVA 800	*Hanseniaspora* spp.	23.0 ± 2	79.5 ± 0.5	32.5 ± 4.5	51.5 ± 0.5	23.5 ± 0.5
DiSVA 605	*H. uvarum*	32.0 ± 2	76.0 ± 2	34.0 ± 8	79.0 ± 8	34.5 ± 0.5
DiSVA 793	*K. unispora*	26.5 ± 0.5	47.0 ± 2	54.0 ± 0	62.0 ± 2	33.0 ± 0
DiSVA 794	*K. unispora*	32.0 ± 2	45.0 ± 0	52.0 ± 2	57.5 ± 0.5	34.0 ± 2
DiSVA 795	*K. unispora*	31.0 ± 2	43.5 ± 0.5	54.0 ± 8	52.5 ± 0.5	26.5 ± 0.5
DiSVA 796	*K. unispora*	24.0 ± 0	47.5 ± 4.5	63.0 ± 2	73.0 ± 8	34.5 ± 4.5
DiSVA 797	*K. unispora*	31.0 ± 2	49.0 ± 2	44.5 ± 0.5	46.0 ± 2	23.5 ± 4.5
DiSVA 798	*K. unispora*	28.5 ± 4.5	44.0 ± 2	41.0 ± 0	52.5 ± 0.5	35.5 ± 0.5
DiSVA 799	*K. unispora*	19.5 ± 4.5	44.5 ± 0.5	60.5 ± 4.5	9.5 ± 0.5	28.0 ± 2
DiSVA 632	*K. marxianus*	24.0 ± 0	49.0 ± 2	40.5 ± 0.5	29.5 ± 0.5	28.5 ± 4.5
DiSVA 634	*K. marxianus*	28.5 ± 4.5	49.0 ± 2	38.5 ± 0.5	18.0 ± 2	22.0 ± 0
DiSVA 322	*L. thermotolerans*	28.5 ± 4.5	46.0 ± 0	41.0 ± 0	0 ± 0	14.5 ± 4.5
DiSVA 323	*L. thermotolerans*	27.0 ± 0	51.5 ± 0.5	49.0 ± 0	69.0 ± 8	23.0 ± 2
DiSVA 324	*L. thermotolerans*	34.0 ± 2	57.5 ± 4.5	35 ± 2	74.5 ± 0.5	41.5 ± 0.5
DiSVA 5	*L. thermotolerans*	27.5 ± 0.5	54.5 ± 0.5	38.0 ± 0	21.0 ± 8	21.5 ± 4.5
DiSVA 325	*L. thermotolerans*	22.0 ± 2	51.5 ± 0.5	40.5 ± 0.5	45.5 ± 0.5	19.0 ± 2
DiSVA 326	*L. thermotolerans*	27.5 ± 0.5	41.5 ± 4.5	28.0 ± 0	25.0 ± 8	7.5 ± 4.5
DiSVA 723	*L. thermotolerans*	19.5 ± 4.5	43.0 ± 2	46.0 ± 8	43.0 ± 2	16.5 ± 4.5
DiSVA 725	*L. thermotolerans*	24.5 ± 0.5	42.5 ± 0.5	44.5 ± 0.5	47.0 ± 8	16.5 ± 0.5
DiSVA 718	*L. waltii*	25.0 ± 2	74.0 ± 2	44.0 ± 8	38.0 ± 2	27.0 ± 0
DiSVA 733	*M. aff. fructicola*	31.5 ± 4.5	52.5 ± 4.5	34.0 ± 2	28.0 ± 2	23.0 ± 2
DiSVA 738	*M. pulcherrima*	28.0 ± 2	58.5 ± 4.5	42 ± 8	30.5 ± 4.5	23.0 ± 2
DiSVA 742	*M. pulcherrima*	36.5 ± 0.5	67.5 ± 4.5	73.0 ± 2	44.0 ± 8	34.0 ± 2
DiSVA 606	*M. pulcherrima*	49.0 ± 2	69.5 ± 0.5	78.0 ± 2	75.0 ± 2	49.5 ± 0.5
DiSVA 269	*M. pulcherrima*	20.0 ± 2	60.5 ± 4.5	33.0 ± 0	37.0 ± 8	29.5 ± 0.5
DiSVA 267	*M. pulcherrima*	37.5 ± 4.5	67.5 ± 0.5	67.0 ± 0	73.0 ± 8	33.0 ± 0
DiSVA 746	*M. reukaufii*	31.0 ± 2	41.5 ± 4.5	43 ± 8	47.5 ± 0.5	20.5 ± 0.5
DiSVA 732	*M. ziziphicola*	34.5 ± 4.5	68.0 ± 2	32.0 ± 2	43.5 ± 4.5	17.0 ± 2
DiSVA 1283	*M. caribbica*	29.0 ± 2	43.5 ± 0.5	37.0 ± 2	36.0 ± 2	21.0 ± 2
DiSVA 1284	*M. caribbica*	28.0 ± 2	62.5 ± 4.5	44.5 ± 0.5	31.0 ± 8	21.0 ± 2
DiSVA 1341	*M. caribbica*	30.5 ± 0.5	73.5 ± 4.5	39.0 ± 2	30.0 ± 2	23.5 ± 0.5
DiSVA 1285	*M. caribbica*	22.0 ± 2	47.5 ± 4.5	40.0 ± 4	30.5 ± 4.5	25.5 ± 4.5
DiSVA 1288	*M. guilliermondii*	24.0 ± 0	53.5 ± 4.5	32.5 ± 4.5	34.0 ± 4	31.5 ± 0.5
DiSVA 1295	*M. guilliermondii*	28.5 ± 4.5	47.5 ± 4.5	37.5 ± 0.5	30.0 ± 2	225 ± 05
DiSVA 1296	*M. guilliermondii*	30.0 ± 0	655 ± 0.5	475 ± 4.5	28.0 ± 2	24.5 ± 0.5
DiSVA 671	*P. anomala*	29.0 ± 2	395 ± 4.5	395 ± 4.5	23.0 ± 0	175 ± 0.5
DiSVA 628	*P. fermentans*	30.5 ± 0.5	49.5 ± 0.5	40.0 ± 8	185 ± 0.5	225 ± 0.5
DiSVA 630	*P. fermentans*	21.0 ± 0	515 ± 0.5	50.0 ± 2	21.5 ± 4.5	20.5 ± 4.5
DiSVA 609	*P. manshurica*	18.5 ± 0.5	485 ± 0.5	25.0 ± 4	15.0 ± 2	11.5 ± 4.5
DiSVA 770	*S. bayanus*	24.5 ± 0.5	505 ± 4.5	43.0 ± 8	40.5 ± 4.5	23 0 ± 2
DiSVA 772	*S. bayanus*	23.5 ± 0.5	65.5 ± 4.5	47.0 ± 2	40.0 ± 8	18.0 ± 0
DiSVA 674	*S. cerevisiae*	25.5 ± 4.5	49.5 ± 4.5	39.5 ± 4.5	42.5 ± 0.5	22.5 ± 0.5
DiSVA 716	*S. cerevisiae*	22.0 ± 2	46.5 ± 4.5	56.0 ± 0	41.5 ± 0.5	18.5 ± 4.5
DiSVA 788	*S. cerevisiae*	27.5 ± 0.5	62.5 ± 4.5	44.5 ± 4.5	56.0 ± 8	29.0 ± 0
DiSVA 860	*S. cerevisiae*	18.5 ± 0.5	82.0 ± 2	50.0 ± 2	61.0 ± 0	24.5 ± 0.5
DiSVA 861	*S. cerevisiae*	24.5 ± 0.5	62.5 ± 0.5	45.0 ± 2	59.5 ± 4.5	25.5 ± 4.5
DiSVA 862	*S. cerevisiae*	22.5 ± 4.5	62.0 ± 2	41.0 ± 0	56.0 ± 2	30.5 ± 4.5
DiSVA 863	*S. cerevisiae*	27.5 ± 0.5	49.5 ± 4.5	37.0 ± 2	30.0 ± 2	21.5 ± 4.5
DiSVA 864	*S. cerevisiae*	30.0 ± 0	78.5 ± 4.5	49.5 ± 0.5	63.0 ± 8	28.5 ± 4.5
DiSVA 789	*S. cerevisiae*	28.5 ± 4.5	81.5 ± 4.5	48.5 ± 0.5	63.0 ± 8	26.5 ± 0.5
DiSVA 790	*S. cerevisiae*	28.5 ± 4.5	67.5 ± 0.5	51.5 ± 0.5	61.5 ± 0.5	26.5 ± 0.5
DiSVA 865	*S. cerevisiae*	31.0 ± 2	76.5 ± 0.5	52.0 ± 2	53.0 ± 8	32.0 ± 2
DiSVA 791	*S. cerevisiae*	28.5 ± 4.5	64 ± 2	36.5 ± 0.5	60.5 ± 0.5	32.0 ± 2
DiSVA 792	*S. cerevisiae*	16.5 ± 4.5	39.0 ± 2	44.0 ± 8	48.5 ± 0.5	21.5 ± 0.5
DiSVA 866	*S. cerevisiae*	25.5 ± 4.5	62.5 ± 0.5	37.0 ± 2	41.0 ± 2	18.5 ± 4.5
DiSVA 769	*S. cerevisiae*	28.0 ± 2	52.5 ± 0.5	37.5 ± 4.5	41.0 ± 8	18.5 ± 4.5
DiSVA 780	*S. cerevisiae*	26.0 ± 2	52.5 ± 0.5	60.0 ± 8	43.0 ± 2	21.0 ± 2
DiSVA 774	*S. cerevisiae*	23.0 ± 2	65.5 ± 4.5	53.0 ± 2	47.0 ± 8	22.0 ± 0
DiSVA 767	*S. cerevisiae*	22.0 ± 2	54.5 ± 0.5	53.0 ± 8	37.0 ± 8	28.0 ± 2
DiSVA 778	*S. cerevisiae*	24.5 ± 0.5	56.0 ± 0	49.0 ± 8	40.5 ± 4.5	22.0 ± 0
DiSVA 766	*S. cerevisiae*	25.5 ± 4.5	53.5 ± 0.5	37.0 ± 2	46.5 ± 4.5	21.5 ± 4.5
DiSVA 764	*S. cerevisiae*	25.5 ± 4.5	48.0 ± 0	37.0 ± 2	41.5 ± 0.5	18.0 ± 0
DiSVA 777	*S. cerevisiae*	25.0 ± 2	46.5 ± 0.5	46.5 ± 0.5	40.0 ± 2	25.5 ± 4.5
DiSVA 763	*S. cerevisiae*	28.5 ± 4.5	57.5 ± 4.5	43.0 ± 2	47.0 ± 2	33.0 ± 0
DiSVA 779	*S. cerevisiae*	27.5 ± 0.5	62.5 ± 4.5	43.0 ± 8	42.0 ± 0	22.5 ± 4.5
DiSVA 776	*S. cerevisiae*	26.5 ± 0.5	58.0 ± 2	45.0 ± 2	45.5 ± 0.5	22.5 ± 0.5
DiSVA 604	*S. cerevisiae*	25.5 ± 4.5	63.5 ± 0.5	46.5 ± 0.5	44.5 ± 0.5	20.5 ± 4.5
DiSVA 719	*S. shehatae*	18.0 ± 0	36.5 ± 4.5	36.0 ± 0	31.0 ± 8	15.5 ± 0.5
DiSVA 720	*S. shehatae*	25.5 ± 4.5	38.5 ± 0.5	38.0 ± 8	21.0 ± 8	15.0 ± 2
DiSVA 734	*S. shehatae*	26.0 ± 2	37.0 ± 0	39.5 ± 4.5	13.5 ± 0.5	17.0 ± 2
DiSVA 426	*T. delbrueckii*	29.0 ± 2	67.5 ± 0.5	49.0 ± 0	42.0 ± 0	23.0 ± 2
DiSVA 399	*T. delbrueckii*	24.0 ± 0	53.5 ± 0.5	43.0 ± 8	39.0 ± 0	26.0 ± 2
DiSVA 413	*T. delbrueckii*	23.0 ± 2	64.5 ± 0.5	41.5 ± 0.5	32.5 ± 0.5	19.5 ± 0.5
DiSVA 602	*T. delbrueckii*	26.0 ± 2	57.5 ± 4.5	37.5 ± 0.5	37.5 ± 4.5	27.5 ± 0.5
DiSVA 419	*T. delbrueckii*	25.5 ± 4.5	63.5 ± 0.5	37.0 ± 2	43.0 ± 2	25.0 ± 2
DiSVA 445	*T. delbrueckii*	22.5 ± 4.5	66.5 ± 0.5	45.5 ± 4.5	47.5 ± 0.5	21.5 ± 0.5
DiSVA 430	*T. delbrueckii*	22.5 ± 4.5	50.5 ± 4.5	48.5 ± 0.5	59.5 ± 4.5	32.5 ± 4.5
DiSVA 432	*T. delbrueckii*	25.0 ± 2	72.5 ± 4.5	39.0 ± 8	50.0 ± 8	27.5 ± 0.5
DiSVA 363	*T. delbrueckii*	20.0 ± 2	50.5 ± 4.5	39.0 ± 2	33.5 ± 4.5	28.0 ± 2
DiSVA 254	*T. delbrueckii*	27.0 ± 0	54.5 ± 0.5	36.0 ± 0	45.5 ± 0.5	24.5 ± 0.5
DiSVA 255	*T. delbrueckii*	23.5 ± 0.5	62.0 ± 2	41.0 ± 0	34.5 ± 0.5	22.5 ± 4.5
DiSVA 256	*T. delbrueckii*	28.5 ± 4.5	50.0 ± 2	51.0 ± 8	39.5 ± 0.5	27.5 ± 4.5
DiSVA 258	*T. delbrueckii*	30.0 ± 0	69.0 ± 2	39.0 ± 8	34.0 ± 2	22.5 ± 0.5
DiSVA 260	*T. delbrueckii*	26.0 ± 2	66.5 ± 0.5	47.5 ± 4.5	43.0 ± 8	23.0 ± 2
DiSVA 261	*T. delbrueckii*	25.0 ± 2	82.5 ± 0.5	83.5 ± 4.5	66.5 ± 4.5	34.5 ± 4.5
DiSVA 313	*T. delbrueckii*	22.0 ± 2	56.5 ± 4.5	52.5 ± 4.5	48.0 ± 0	31.5 ± 4.5
DiSVA 315	*T. delbrueckii*	27.0 ± 0	54.5 ± 0.5	49.5 ± 0.5	33.5 ± 4.5	23.0 ± 2
DiSVA 55	*T. delbrueckii*	24.0 ± 0	62.5 ± 0.5	37.5 ± 4.5	59.5 ± 4.5	25.5 ± 4.5
DiSVA 343	*T. delbrueckii*	23.5 ± 0.5	47.5 ± 4.5	42.5 ± 4.5	44.0 ± 2	32.0 ± 2
DiSVA 130	*T. delbrueckii*	24.5 ± 0.5	65.5 ± 4.5	51.0 ± 0	57.5 ± 0.5	22.5 ± 4.5
DiSVA 671	*W. anomalus*	23.5 ± 0.5	38.5 ± 0.5	36.0 ± 0	21.5 ± 4.5	17.5 ± 0.5
DiSVA 2	*W. anomalus*	24.0 ± 0	45.0 ± 2	56.0 ± 4	21.0 ± 3	18.5 ± 0.5
Control	*A. pullulans*	29.5 ± 0.5	74.5 ± 4.5	15.0 ± 4	49.0 ± 2	11.5 ± 4.5
Control	*S. boulardii*	23.0 ± 1	46.5 ± 0.5	27.0 ± 2	25.5 ± 0.5	27.0 ± 0

**Fig. 2:**
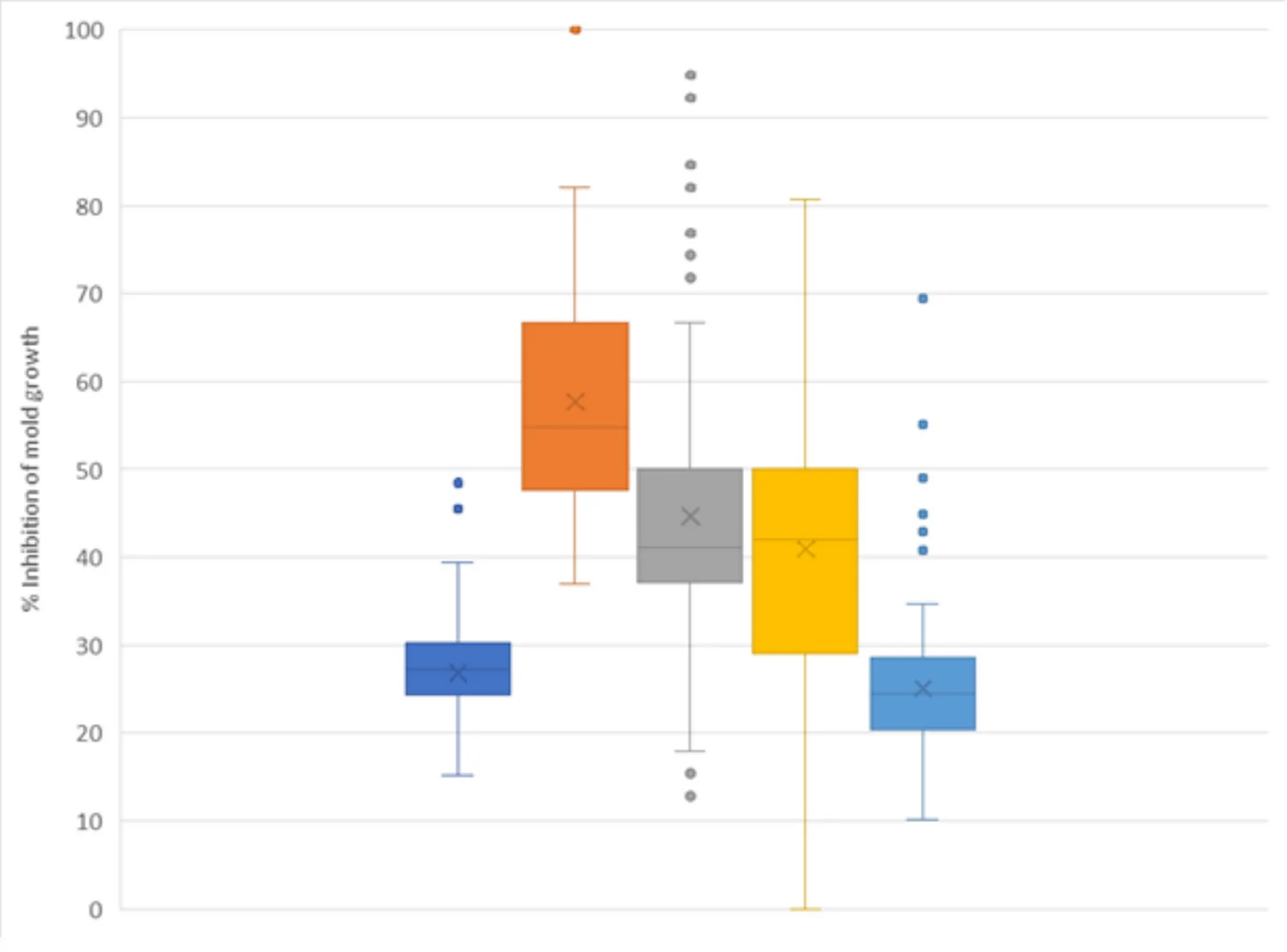
Box plot shows the distribution of the percentage reduction growth of *P. expansum* (  ), *B. cinerea* (  ), *P. digitatum* (  ), *Cladosporium* spp. (  ), and *A. carbonarius* (  ) by yeast activity

A combined evaluation of Table 3 and Fig. 2 (box plots) showed clear differences among molds. For *P. expansum*, 29 strains showed inhibition values above the third quartile (Q3), ranging from 30.3% to 48.5% and representing the top-performing fraction of the dataset. Within this group, two outliers were identified: *B. bruxellensis* DiSVA 810 and *M. pulcherrima* DiSVA 606, both achieving 48.5% inhibition.

*B. cinerea* exhibited the highest susceptibility, with 29 strains above Q3 (inhibition values from 66.7% to 100%). A notable outlier was *B. bruxellensis* DiSVA 810, which achieved complete inhibition (Table 3). A similar but more variable pattern was observed for *P. digitatum*, where 27 strains showed inhibition values above Q3, ranging from 51.3% to 94.9%. Several high-performing outliers were identified, including *B. bruxellensis* DiSVA 810, DiSVA 812, and DiSVA 816, with a mean inhibition of 92.3%, *T. delbrueckii* DiSVA 261 (82.1%), and *M. pulcherrima* DiSVA 606 (76.2%). In contrast, *Cladosporium* spp. showed a more homogeneous distribution, with 27 strains above Q3 and inhibition values between 51.6% and 80.7%, without evident outliers, although the highest inhibition was observed for DiSVA 605 (80.7%). Finally, *A. carbonarius* was the least sensitive species, with 28 strains above Q3, corresponding to inhibition values ranging from 28.6% to 69.4%. However, six clear outliers were identified, including *B. bruxellensis* DiSVA 810, DiSVA 812, DiSVA 814, and DiSVA 816, with a mean inhibition of 43.9%, *M. pulcherrima* DiSVA 606 (49.9%), and *A. pullulans* DiSVA 211 (64.4%), all showing markedly higher inhibition compared to the rest of the dataset.

Overall, the inhibitory effect of yeasts varied markedly among different mold species. *B. cinerea* showed the highest sensitivity, with a median inhibition of 54.8% with several yeast strains that exceeding 80%, indicating a strong and occasionally complete growth suppression. The relatively wide interquartile range reflects moderate variability among replicates. *P. digitatum* and *Cladosporium* spp. exhibited intermediate susceptibility with a median inhibition values of 41.0 and −41.9% respectively, with a broad distribution of data points, suggesting heterogeneous responses to the treatment. Notably, both species included low and high extremes, indicating strain-dependent effects.

*P. expansum* displayed the lowest susceptibility profile, with a median of 27.3% and a slighter dispersion, indicating a more consistent but limited reduction in growth.

*A. carbonarius* was the least affected species, showing a median inhibition of 24.5%. Higher inhibition levels (> 50%) were mainly observed against *B. cinerea* (73 strains), followed by *P. digitatum* (27 strains) and *Cladosporium* spp. (27 strains), whereas *A. carbonarius* was the least affected, with only 2 strains showing inhibition above 50%. Although some higher inhibition values were observed, most data clustered at lower percentages, suggesting relative resistance compared to the other molds.

In summary, the results demonstrated species-dependent antifungal activity, with *B. cinerea* being the most susceptible and *A. carbonarius* the most resistant, while *P. digitatum* and *Cladosporium* spp. showed intermediate and variable responses.

**Fig. 3:**
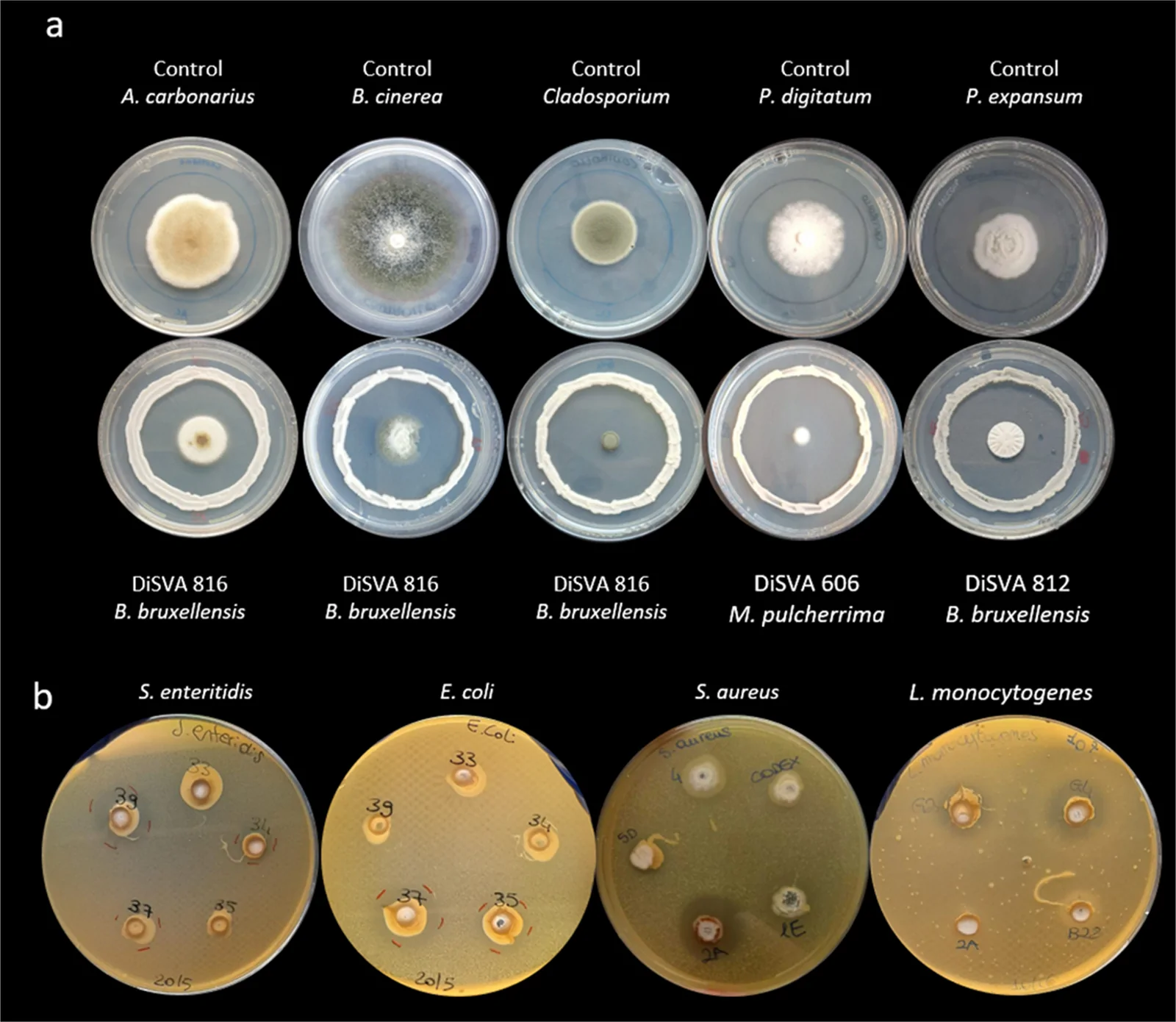
Inhibitory activity of selected yeast strains against fungal and bacterial pathogens. **a** Anti-mold activity produced by selected yeast strains against *A. carbonarius*, *B. cinerea*, *Cladosporium* spp., *P. digitatum*, and *P. expansum*. The upper row shows control plates (mold grown in the absence of yeast), while the lower row shows mold growth in the presence of yeast strains (indicated below each plate). Growth reduction indicates the antifungal effect of yeast. **b** Antibacterial activity produced by selected yeast strains against *S. enteritidis*, *E. coli*, *S. aureus*, and *L. monocytogenes*. Clear inhibition zones around the wells indicate growth suppression of the target bacteria. Yeast strains on plate are reported on plate with their internal code. From left to right: the *S. enteritidis* plate tested against five strains of *T. delbrueckii* (DiSVA 254, DiSVA 602, DiSVA 256, DiSVA 258, and DiSVA 260); the *E. coli* plate tested against five strains of *T. delbrueckii* (DiSVA 254, DiSVA 602, DiSVA 256, DiSVA 258, and DiSVA 260); the *S. aureus* plate tested against 2 A (DiSVA 606), 5D (DiSVA 609), 4 (DiSVA 776), the control strain CODEX, and 1E (DiSVA 605); and the *L. monocytogenes* plate tested against G2 (DiSVA 810), G4 (DiSVA 812), 2 A (DiSVA 606), and B28 (DiSVA 733)

Figure 3 shows some representative photos of the results of the plate tests relating to the antibacterial and antifungal activities performed by the yeasts tested in comparison with the controls.

### Antibacterial activity mediated by VOCs

After preliminary screening, yeast strains were selected based on their antibacterial performance, particularly those showing broad-spectrum activity against at least three pathogenic bacteria. For the anti-mold assays, only strains with inhibition values above the third quartile of the boxplot distribution (Fig. 2) were considered. The datasets obtained from antibacterial, anti-mold, and killer activity assays were then cross-compared and 21 yeast strains showing the highest overall inhibitory activity against the different tested microorganisms (molds, yeasts, and bacteria) were selected for further investigation of their biocontrol potential.

**Fig. 4:**
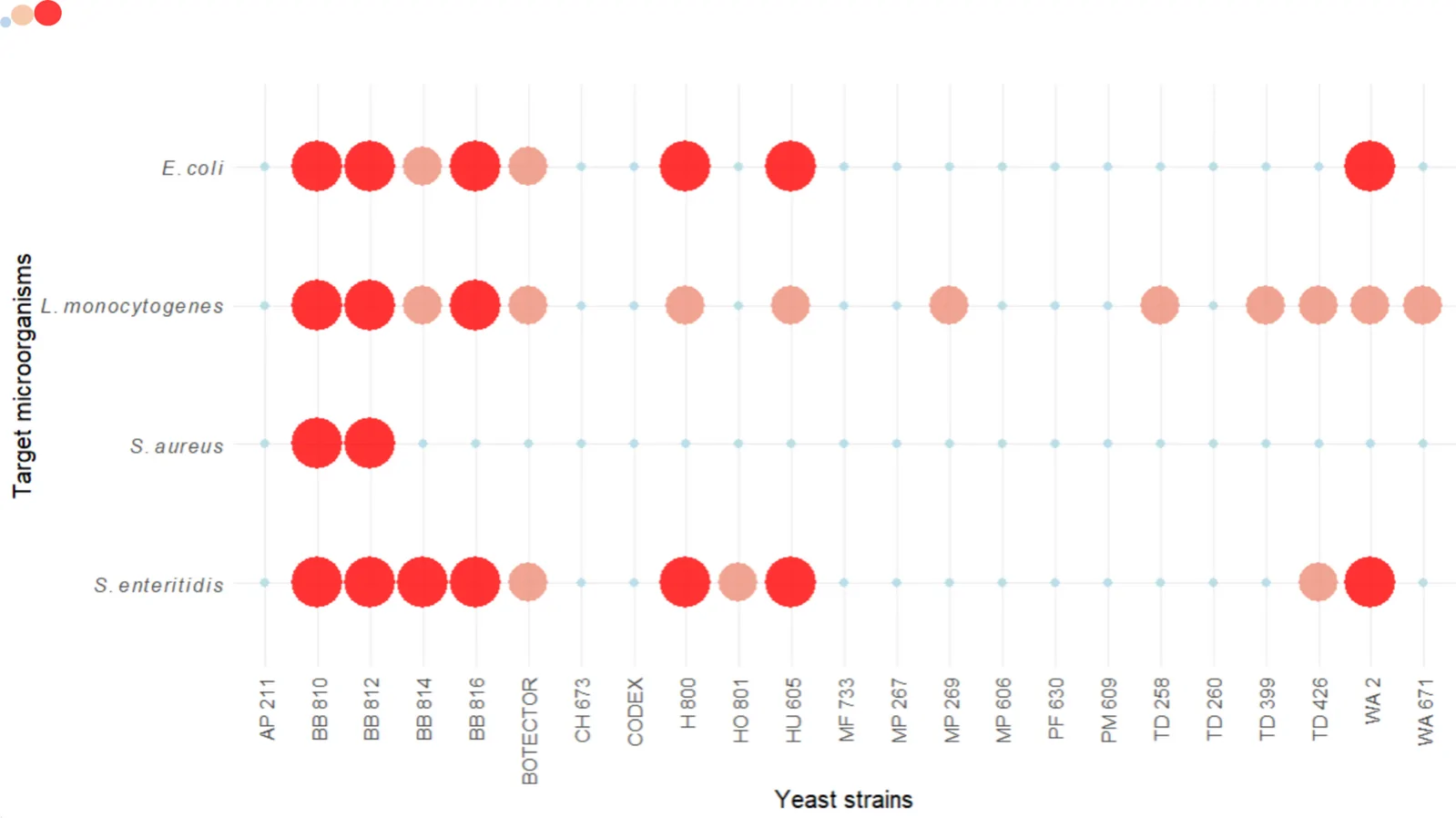
VOC-mediated inhibitory activity of selected yeast strains against *E. coli, S. aureus, S. enteritidis* and *L. monocytogenes*. Both colour and bubble size represent the level of inhibition: no inhibition (  ), partial inhibition (  ), total inhibition (  ). On *x*-axis, yeast strains are labelled using the species initials followed by the corresponding DiSVA strain number

Following the preliminary screening, a two-step approach combining statistical filtering and functional prioritization was applied to select the best-performing strains. First, for each fungal pathogen, strains with inhibition values exceeding the third quartile (Q3) were identified, representing the top 25% of isolates. This threshold served as a statistical guideline to highlight the most promising antifungal candidates. Since this approach yielded a relatively large number of strains, a second selection criterion based on antibacterial activity was applied. Specifically, priority was given to strains exhibiting broad-spectrum activity, defined as the ability to inhibit at least three bacterial targets. The final panel of 21 strains was established by integrating these criteria, prioritizing those that consistently demonstrated both strong antifungal performance and broad antibacterial activity. Consequently, while the Q3 threshold provided the primary statistical filter, additional biologically relevant factors (i.e. spectrum of activity) were used to refine the final selection. This subset included the most active isolates from the previous screenings: *B. bruxellensis* (DiSVA 810, DiSVA 812, DiSVA 814, DiSVA 816), *M. fructicola* (DiSVA 733), *M. pulcherrima* (DiSVA 606, DiSVA 267, DiSVA 269), *H. osmophila* (DiSVA 801), *Hanseniaspora* spp. (DiSVA 800), *H. uvarum* (DiSVA 605), *T. delbrueckii* (DiSVA 426, DiSVA 399, DiSVA 258, DiSVA 260)* W. anomalus* (DiSVA 2, DiSVA 671), *P. fermentans* (DiSVA 630), and *P. manshurica* (DiSVA 609). Accordingly, the contribution of volatile organic compounds (VOCs) to the observed antimicrobial profiles was evaluated. The statistical analysis was carried out using Mann-Whitney *U*-test to validate this approach since the selected panel showed a significant higher antimicrobial activity than non-selected strains apart from three mold tests which however showed a superior activity (Supplementary Figs. 1). Figure 4 shows the inhibitory activity mediated by volatile organic compounds (VOCs) produced by selected yeast strains against the same enteropathogenic bacteria tested (*E. coli*, *S. aureus*, *S. enteritidis*, and *L. monocytogenes*)*.*

In general, the most pronounced inhibitory effects under the experimental conditions tested were observed against *E. coli* and *S. enteritidis*. No visible macroscopic growth was detected for these bacteria when exposed to specific yeast isolates, notably those belonging to the genus *Brettanomyces* (DiSVA 810, DiSVA 812, DiSVA 814, DiSVA 816). These strains exhibited a promising inhibitory potential, suggesting a volatile-mediated antagonism that merits further investigation through chemical characterization to definitively identify the bioactive compounds involved.

The strains DiSVA 810 and DiSVA 812 were the only ones for which no visible growth was observed across all four bacterial strains.

This suggests that these strains produce VOCs with a broad range of antimicrobial activity. *W. anomalus* (DiSVA 2), *H. osmophila* (DiSVA 801), and *M. fructicola* (DiSVA 733) strains also showed remarkable inhibition, effectively reducing the growth of multiple target bacteria strains with the exclusion of *S. aureus*.

Overall, the results highlighted *Brettanomyces* and to a lesser extent *W. anomalus* and *H. osmophila* as the most effective VOC-producing yeasts against enteropathogenic bacteria, whereas other antimicrobial active yeasts may rely mainly on non-volatile metabolites or direct-contact mechanisms to exert their antimicrobial effects.

### Antifungal activity mediated by VOCs

**Fig. 5:**
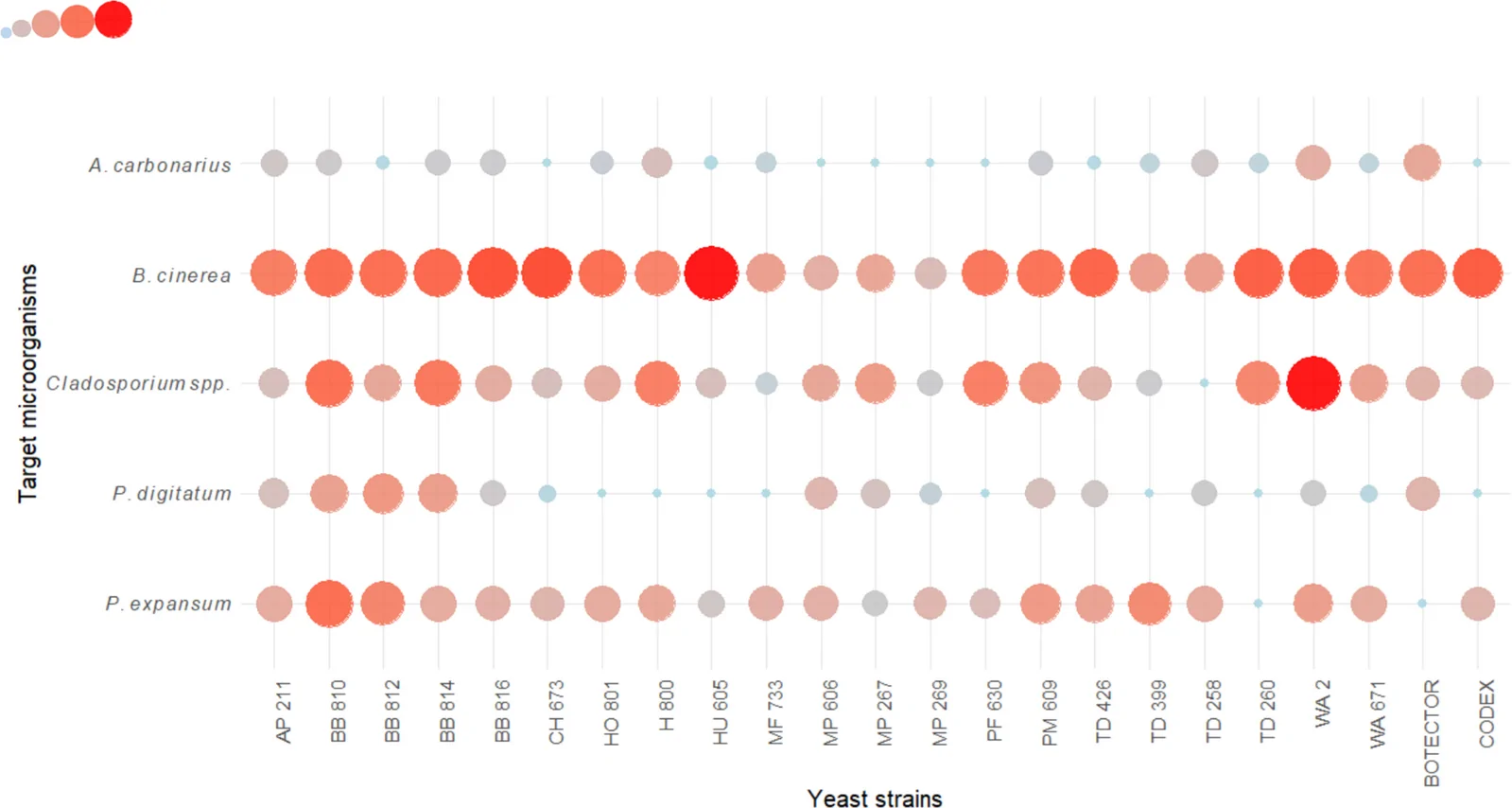
VOC-mediated inhibitory activity of tested yeast strains against *A. carbonarius, B. cinerea, P. digitatum, P. expansum and Cladosporium* spp. Both colour intensity and bubble size are proportional to the percentage of reduction in fungal growth: 0% inhibition (  ), 25% inhibition (  ), 50% inhibition (  ), 75% inhibition (  ), and 100% inhibition (  ). On *x*-axis, yeast strains are labelled using the species initials followed by the corresponding DiSVA strain number

As reported in Fig. 5, among the fungal strains, *B. cinerea* emerged as the most sensitive species to the volatile compounds produced by yeasts, followed by *Cladosporium* spp., which also showed marked but more variable inhibition. In contrast, *P. expansum*, *P. digitatum*, and *A. carbonarius* were generally less affected by VOCs, with limited or no inhibition observed for most yeast strains, except for a few active strains.

Among these, *Brettanomyces* strains (DiSVA 810, DiSVA 812, DiSVA 814, DiSVA 816) confirmed their strong inhibitory potential through VOCs emission, consistent with the results obtained against pathogens bacteria. Similarly, *W. anomalus* (DiSVA 2, DiSVA 671), *Hanseniaspora* spp. (DiSVA 801, DiSVA 800, DiSVA 605), and *Pichia manshurica* (DiSVA 609) exhibited high inhibition levels against multiple fungal targets, reaching up to 100% reduction in the case of *Cladosporium* spp. Moderate inhibition was observed for some *Torulaspora* and *A. pullulans* strains, which displayed partial but significant VOCs-mediated effects. Conversely, strains of *M. pulcherrima* showed limited VOCs production, suggesting that their antifungal action may rely mainly non-volatile metabolites or direct-contact mechanisms rather than on VOCs emission. After a preliminary statistical evaluation of the results of VOCs by Anova and Duncan’s test (Supplementary Table s1), principal component analysis (PCA) was conducted.

**Fig. 6:**
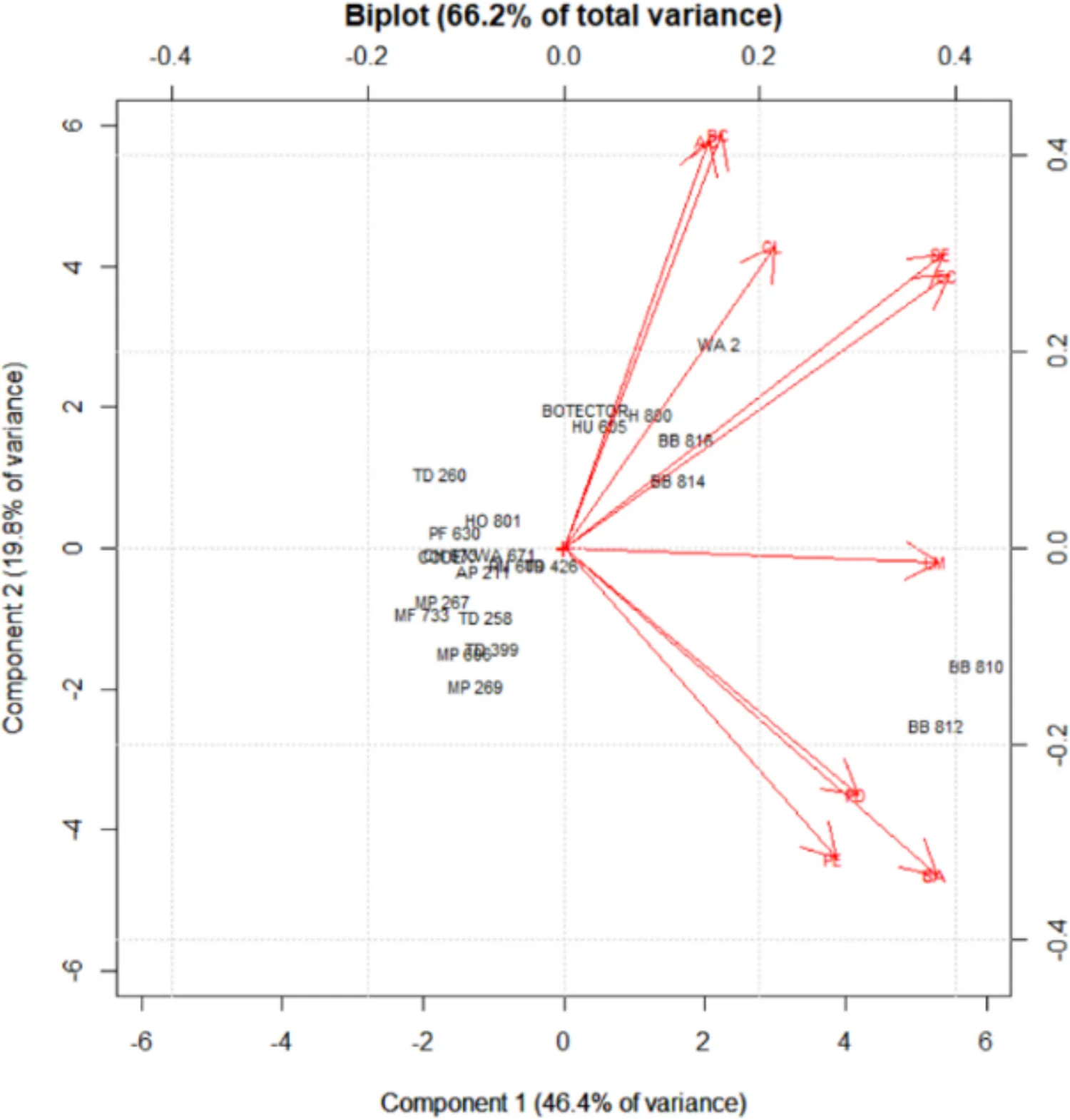
Principal component analysis (PCA) of VOC-mediated antimicrobial effects**.** The variance explained by PCA is PC 1 = 46.4% on *x*-axis and PC 2 = 19.8% on *y*-axis

The PCA biplot analysis showed that the first two principal components explained 66.2% of the total variance, with PC1 accounting for 46.4% and PC2 for 19.8% of variance (Fig. 6). PC1 primarily separated yeast strains according to the overall level of VOC-mediated antimicrobial activity, whereas PC2 further differentiates strains based on the distribution of activity across target microorganisms.

The vectors, corresponding to VOCs activity against *E. coli*, *S. enteritidis*, *L. monocytogenes*, and *S. aureus*, are closely aligned, indicating a strong positive correlation among antibacterial VOCs effects. In contrast, vectors associated with antifungal activity are oriented along a different direction, contributing mainly to separation along PC2.

*Brettanomyces* strains (DiSVA 810, DiSVA 812, DiSVA 814, DiSVA 816) are clearly separated from the other yeasts and positioned on the positive quadrants of PC1, showing strong associations with both antibacterial and antifungal VOC variables. The greatest action was displayed by DiSVA 810 and DiSVA 812 strains. Other strains, including *W. anomalus* (DiSVA 2) and *Hanseniaspora* (DiSVA 605, DiSVA 800), showed intermediate positions, reflecting moderate VOC-mediated antimicrobial activity. In contrast, strains belonging to *Metschnikowia*, *Saccharomyces*, and *A. pullulans* cluster near the origin, indicating low or negligible VOC-mediated antimicrobial effects.

Overall, the PCA highlights a clear separation between yeast strains with strong VOC-mediated antimicrobial activity and those with limited or no volatile-associated effects, as well as variability among genera, species and strains.

## Discussion

Microbial communities are characterized by a wide range of interactions, from mutualistic and commensal relationships to competitive and antagonistic behaviours. Yeasts have been investigated mainly in applied and industrial contexts, particularly as fermentative organisms or as biological control agents (BCAs) against postharvest fungal pathogens (Danquah et al. 2022). Their role as sources of antimicrobial activity against pathogenic bacteria has received comparatively less attention, and they are often considered secondary contributors to antimicrobial interactions when compared with bacteria or filamentous fungi.

Nevertheless, considerable evidence indicated that yeasts could exhibit inhibitory effects through multiple mechanisms, such as competition for nutrients and space, secretion of diffusible antimicrobial metabolites, production of killer toxins, iron sequestration, and the release of volatile organic compounds (VOCs) (Arrarte et al. 2017). The coexistence of such diverse mechanisms suggests that yeast-microorganism interactions are highly context-dependent and strongly influenced by strain-specific traits, environmental conditions, and microbial community structure (Querol and Fleet 2006).

In the present study, a wide and taxonomically assorted collection of yeast strains was evaluated for antimicrobial activity against enteropathogenic bacteria and plant pathogens as well as spoilage molds using both direct-contact and VOC-mediated assays.

The antimicrobial activity of several strains identified in this study is consistent with previously reported data. *Metschnikowia* spp., and particularly *M. pulcherrima*, showed a broad inhibitory spectrum against both molds and bacteria but this action was not strictly associated with VOC-mediated activity. This observation indicates that the antimicrobial activity of *Metschnikowia* is mainly driven by non-volatile mechanisms. Indeed, several studies reported the antifungal activity of *M. pulcherrima* against molds such as *B. cinerea* and *Monilinia laxa* (Ruiz-Moyano et al. 2016; Oro et al. 2018), as well as its inhibitory effects against spoilage yeasts during wine fermentation (Oro et al. 2014). These antagonistic properties have been attributed to multiple factors, including pulcherrimin-mediated iron chelation, killer toxin production, and the secretion of extracellular antimicrobial metabolites (Oro et al. 2014; Sipiczki 2020; Büyüksırıt-Bedir and Kuleaşan 2022). The results obtained in the present study are consistent with this multifactorial model and further support the idea that different yeast genera rely on fundamentally distinct antimicrobial strategies. The variability observed in antibacterial activity among *Metschnikowia* strains is also in line with previous reports describing strain-dependent production of antimicrobial proteins and metabolites (Hicks et al. 2021).

The genera *Hanseniaspora* and *Wickerhamomyces* exhibited a more versatile inhibitory behaviour. Two *Hanseniaspora* strains (DiSVA 800, DiSVA 605) were characterized by a broad-spectrum antibacterial activity mediated by VOC production. These findings are supported by several studies reporting antifungal activity of different *Hanseniaspora* species, including *H. uvarum*, *H. guillermondii*, and *H. opuntiae*, against molds such as *Aspergillus* spp., *Cladosporium* spp., and *Botrytis* spp. (Cordero-Bueso et al. 2017; Fernández‐Pacheco et al. 2021). Current evidence indicates that *Hanseniaspora* spp. primarily act as biocontrol agents through multiple mechanisms, including killer activity (Nandhini et al. 2021), production of antifungal VOCs (Liu et al. 2014; Guo et al. 2019), and induction of host defence responses (Wang et al. 2019). While their antifungal properties are well documented, antibacterial activity remains largely unexplored, making the bacterial inhibition observed in this study particularly noteworthy.

Similarly, *Wickerhamomyces* spp. have been reported to exhibit both antifungal activity against molds such as *P. expansum* and *B. cinerea* (Coda et al. 2011; Oro et al. 2018) and antibacterial activity, including inhibition of *Acinetobacter baumannii* (Junges et al. 2020). The mechanisms proposed for this genus are different and include the production of VOCs such as ethyl acetate and other low-molecular-weight alcohols and esters (Guo et al. 2025), which are considered major contributors to postharvest disease suppression. In addition, antimicrobial activity has been associated with the production of proteinaceous toxins targeting cell wall components (Fernández De Ullivarri et al. 2018). Together, these observations indicate that *Hanseniaspora* and *Wickerhamomyces* combine strong antifungal activity with a broader antimicrobial potential. Several other genera, including *Kazachstania*, *Torulaspora*, *Meyerozyma*, *Lachancea*, *Kluyveromyces*, *Candida*, and *Aureobasidium*, have been widely described as effective biocontrol agents against filamentous fungi, particularly in postharvest environments (Di Francesco et al. 2021). Their antagonistic activity has mainly been linked to antifungal mechanisms, whereas antibacterial effects have been reported less frequently. In agreement with these observations, strains belonging to these genera in the present study showed a prevalence of antifungal activity, while antibacterial effects were limited or strongly strain-dependent under tested conditions. This suggests that although antibacterial inhibition may occur in specific strains, antifungal activity represents the dominant antagonistic behaviour for these yeasts.

*A. pullulans* provide a clear example of this functional specialization. This species has been widely recognized as a biocontrol agent against major postharvest fruit pathogens and as a sustainable alternative to synthetic fungicides (Di Francesco et al. 2018; Cignola et al. 2023, 2024). Consistently, the *A. pullulans* DiSVA 211 analyzed in this study displayed exclusively antifungal activity, mediated by both direct-contact and VOC-based interactions, with no detectable antibacterial effects. Similarly, species belonging to the genera *Meyerozyma* have been reported as effective biocontrol agents against *Aspergillus ochraceus* on grapes and *P. expansum* on kiwifruit (Qiu et al. 2022; Wu et al. 2025). In line with these reports, *Meyerozyma* strains in the present work showed more consistent antifungal than antibacterial activity, although some degree of strain-to-strain variability was observed.

Preliminary observations from this study highlight the antimicrobial trends of the *B. bruxellensis* strains evaluated, which displayed a broad, albeit strain-dependent, inhibitory profile. Although *B. bruxellensis* has been widely studied for its technological relevance and, more recently, for its probiotic potential, particularly in fermented beverages such as kombucha tea (Areal-Hermida et al. 2024), its antimicrobial properties were not investigated. All *B. bruxellensis* strains tested inhibited both filamentous fungi and enteropathogenic bacteria, with some isolates (DiSVA 810, DiSVA 812) showing no visible growth across all bacterial targets. Importantly, this activity was largely mediated by VOCs, indicating that volatile metabolites play a central role in the antagonistic strategy of this specie. This finding suggests that *B. bruxellensis* may rely on VOC production as a specialized ecological adaptation to outcompete rival microorganisms in nutrient-limited environments. Beyond its ecological significance, these results highlight the untapped biotechnological potential of this species as a robust source of antimicrobial volatiles. Leveraging these yeast-derived volatiles could provide a sustainable, chemical-free alternative for extending the shelf life of perishable goods, effectively transforming a traditionally ‘spoilage’ microorganism into a valuable biological tool for food biopreservation. However, further investigations conducted on the identification of antimicrobial compounds and in real food system chain are needed. Overall, antimicrobial activity appeared to be species/genera-specific with a wide variability strain-dependent. When the data were considered at a broader level, recurring patterns of antagonistic behaviour emerged, allowing the identification of genera with relatively consistent inhibitory profiles such as *Brettanomyces* genera.

Taken together, these results highlight the complexity of yeast-mediated antimicrobial interactions and indicate that yeasts cannot be simply classified as antifungal or antibacterial agents. Instead, their inhibitory potential reflects a combination of genera-level tendencies, strain-specific traits, target microorganisms, and interaction mechanisms. Although the present study is limited to in vitro conditions and did not identify the chemical nature of the active compounds, it provides a broad comparative framework that expands current knowledge of yeast antimicrobial activity and supports the inclusion of yeasts as promising and still underexplored candidates for the development of sustainable antimicrobial and biocontrol strategies.

## Conclusion

Controlling microbial contamination remains a critical challenge for ensuring food safety and shelf life. This study highlights yeast diversity as a promising source of antimicrobial traits and identifies candidate strains for further mechanistic and applied investigation. Yeasts have increasingly attracted attention as potential biocontrol agents due to their capacity to interfere with the growth of spoilage and pathogenic microorganisms through multiple mechanisms. This study suggests that specialized metabolic traits, including killer activity and interactions possibly mediated by volatile compounds, might be more widespread among food-associated yeasts than previously thought by expanding our understanding of yeast biodiversity as a source of antimicrobial activity.

Some strains exhibited a wide inhibitory spectrum and showed the ability to affect microbial growth through mechanisms that may involve volatile-mediated interactions. The antimicrobial activity was conducted under standardized laboratory conditions; we recognize that the food matrix complexity (e.g. nutrient availability, pH, and microstructure) can significantly influence the potential role of VOCs. Moreover, additional experiments (e.g. basic metabolite profiling) are needed to future possible application in food systems.

These findings contribute to expanding the knowledge on yeast biodiversity as a source of antimicrobial activity and support the potential use of selected yeasts as candidates for innovative biocontrol strategies in food systems.

## Supplementary Information

Below is the link to the electronic supplementary material.

ESM 1 (DOCX 1.18 MB)

## Data Availability

Data will be made available on request.
