# Supplementary material for: Evaluating yeasts as biocontrol agents against pathogenic and spoilage microorganisms in agri-food: action of volatile organic compounds (VOCs)
Source: Appl Microbiol Biotechnol. 2026 Jun 6;110:244. doi: 10.1007/s00253-026-13896-w (PMC13461708; doi:10.1007/s00253-026-13896-w)
Supplement: Supplementary file 1 [file 253_2026_13896_MOESM1_ESM.docx]

**Supplementary**

**Figure s1.** Boxplots of antimicrobial and antimold activity of selected (n = 19) used for VOCs activity and non-selected (n = 89) strains. Data are shown as median and interquartile range. Statistical differences were assessed using the Mann–Whitney U test in RStudio. with p-values reported in each panel; p < 0.05 was considered statistically significant.

|  | **E. coli** | | **S. aureus** | | **S. enteritidis** | | **L. monocytogenes** | | **A. carbonarius** | | **B. cinerea** | | **P. expansum** | | **P. digitatum** | | **Cladosporium** | |
| --- | --- | --- | --- | --- | --- | --- | --- | --- | --- | --- | --- | --- | --- | --- | --- | --- | --- | --- |
| **DiSVA 211** | 0.0 ± 0.0 | ^c^ | 0.0 ± 0.0 | ^b^ | 0.0 ± 0.0 | ^c^ | 0.0 ± 0.0 | ^c^ | 15.9 ± 0.0 | ^d^ | 66.3 ± 0.2 | ^j^ | 36.2 ± 1.1 | ^h^ | 20.0 ± 2.00 | ^f^ | 20.8 ± 1.4 | ^m^ |
| **DiSVA 810** | 100.0 ± 0.0 | ^a^ | 100.0 ± 0.0 | ^a^ | 100.0 ± 0.0 | ^a^ | 100.0 ± 0.0 | ^a^ | 13.7 ± 1.1 | ^e^ | 74.9 ± 1.8 | ^g^ | 73.6 ± 0.7 | ^a^ | 44.2 ± 1.13 | ^c^ | 72.9 ± 1.8 | ^b^ |
| **DiSVA 812** | 100.0 ± 0.0 | ^a^ | 100.0 ± 0.0 | ^a^ | 100.0 ± 0.0 | ^a^ | 100.0 ± 0.0 | ^a^ | 0.7 ± 0.9 | ^ij^ | 74.0 ± 0.0 | ^gh^ | 61.1 ± 0.1 | ^b^ | 50.5 ± 0.50 | ^a^ | 38.5 ± 0.6 | ^i^ |
| **DiSVA 814** | 50.0 ± 0.0 | ^b^ | 0.0 ± 0.0 | ^b^ | 100.0 ± 0.0 | ^a^ | 50.0 ± 0.0 | ^b^ | 12.0 ± 2.0 | ^f^ | 77.4 ± 0.3 | ^f^ | 36.2 ± 1.1 | ^h^ | 46.1 ± 1.62 | ^b^ | 69.8 ± 0.1 | ^c^ |
| **DiSVA 816** | 100.0 ± 0.0 | ^a^ | 0.0 ± 0.0 | ^b^ | 100.0 ± 0.0 | ^a^ | 100.0 ± 0.0 | ^a^ | 14.0 ± 2.0 | ^e^ | 87.5 ± 0.5 | ^b^ | 31.6 ± 0.8 | ^i^ | 15.2 ± 1.13 | ^g^ | 34.9 ± 0.0 | ^j^ |
| **DiSVA 673** | 0.0 ± 0.0 | ^c^ | 0.0 ± 0.0 | ^b^ | 0.0 ± 0.0 | ^c^ | 0.0 ± 0.0 | ^c^ | 0.0 ± 0.0 | ^j^ | 88.4 ± 0.0 | ^b^ | 29.5 ± 0.5 | ^j^ | 4.1 ± 1.62 | ^i^ | 22.3 ± 0.8 | ^m^ |
| **DiSVA 800** | 100.0 ± 0.0 | ^a^ | 0.0 ± 0.0 | ^b^ | 100.0 ± 0.0 | ^a^ | 50.0 ± 0.0 | ^b^ | 21.3 ± 0.2 | ^c^ | 62.5 ± 0.5 | ^k^ | 39.3 ± 0.8 | ^g^ | 0.0 ± 0.0 | ^j^ | 66.1 ± 1.6 | ^d^ |
| **DiSVA 801** | 0.0 ± 0.0 | ^c^ | 0.0 ± 0.0 | ^b^ | 50.0 ± 0.0 | ^b^ | 0.0 ± 0.0 | ^c^ | 11.0 ± 1.8 | ^f^ | 74.0 ± 0.0 | ^gh^ | 36.2 ± 1.1 | ^h^ | 0.0 ± 0.0 | ^j^ | 35.4 ± 0.7 | ^j^ |
| **DiSVA 605** | 100.0 ± 0.0 | ^a^ | 0.0 ± 0.0 | ^b^ | 100.0 ± 0.0 | ^a^ | 50.0 ± 0.0 | ^b^ | 1.7 ± 0.2 | ^ij^ | 100.0 ± 2.0 | ^a^ | 17.0 ± 1.8 | ^m^ | 0.0 ± 0.0 | ^j^ | 22.3 ± 0.8 | ^m^ |
| **DiSVA 733** | 0.0 ± 0.0 | ^c^ | 0.0 ± 0.0 | ^b^ | 0.0 ± 0.0 | ^c^ | 0.0 ± 0.0 | ^c^ | 7.6 ± 0.3 | ^g^ | 43.7 ± 0.9 | ^l^ | 31.6 ± 0.8 | ^i^ | 0.0 ± 0.0 | ^j^ | 7.8 ± 1.4 | ^o^ |
| **DiSVA 267** | 0.0 ± 0.0 | ^c^ | 0.0 ± 0.0 | ^b^ | 0.0 ± 0.0 | ^c^ | 0.0 ± 0.0 | ^c^ | 0.0 ± 0.0 | ^j^ | 39.8 ± 1.4 | ^m^ | 11.9 ± 1.8 | ^n^ | 19.2 ± 0.08 | ^f^ | 46.9 ± 1.6 | ^g^ |
| **DiSVA 269** | 0.0 ± 0.0 | ^c^ | 0.0 ± 0.0 | ^b^ | 0.0 ± 0.0 | ^c^ | 50.0 ± 0.0 | ^b^ | 0.0 ± 0.0 | ^j^ | 25.0 ± 1.8 | ^o^ | 26.7 ± 0.9 | ^k^ | 27.7 ± 0.18 | ^de^ | 14.0 ± 2.0 | ^n^ |
| **DiSVA 606** | 0.0 ± 0.0 | ^c^ | 0.0 ± 0.0 | ^b^ | 0.0 ± 0.0 | ^c^ | 0.0 ± 0.0 | ^c^ | 0.0 ± 0.0 | ^j^ | 33.1 ± 0.1 | ^n^ | 31.6 ± 0.8 | ^i^ | 27.2 ± 0.08 | ^e^ | 38.5 ± 0.6 | ^i^ |
| **DiSVA 630** | 0.0 ± 0.0 | ^c^ | 0.0 ± 0.0 | ^b^ | 0.0 ± 0.0 | ^c^ | 0.0 ± 0.0 | ^c^ | 0.0 ± 0.0 | ^j^ | 69.7 ± 0.9 | ^i^ | 21.8 ± 1.2 | ^l^ | 0.0 ± 0.0 | ^j^ | 66.1 ± 1.6 | ^d^ |
| **DiSVA 609** | 0.0 ± 0.0 | ^c^ | 0.0 ± 0.0 | ^b^ | 0.0 ± 0.0 | ^c^ | 0.0 ± 0.0 | ^c^ | 10.8 ± 1.3 | ^f^ | 72.1 ± 0.0 | ^h^ | 47.7 ± 0.9 | ^d^ | 20.0 ± 2.00 | ^f^ | 53.1 ± 1.6 | ^f^ |
| **DiSVA 258** | 0.0 ± 0.0 | ^c^ | 0.0 ± 0.0 | ^b^ | 0.0 ± 0.0 | ^c^ | 50.0 ± 0.0 | ^b^ | 15.9 ± 0.0 | ^d^ | 43.7 ± 0.9 | ^l^ | 36.2 ± 1.1 | ^h^ | 12.4 ± 0.41 | ^h^ | 0.0 ± 0.0 | ^p^ |
| **DiSVA 260** | 0.0 ± 0.0 | ^c^ | 0.0 ± 0.0 | ^b^ | 0.0 ± 0.0 | ^c^ | 0.0 ± 0.0 | ^c^ | 4.9 ± 1.6 | ^h^ | 81.7 ± 0.1 | ^d^ | 0.0 ± 0.0 | ^o^ | 0.0 ± 0.0 | ^j^ | 61.4 ± 0.6 | ^e^ |
| **DiSVA 399** | 0.0 ± 0.0 | ^c^ | 0.0 ± 0.0 | ^b^ | 0.0 ± 0.0 | ^c^ | 50.0 ± 0.0 | ^b^ | 5.4 ± 0.3 | ^h^ | 45.2 ± 1.2 | ^l^ | 58.5 ± 0.40 | ^c^ | 0.0 ± 0.0 | ^j^ | 12.50± 0.5 | ^n^ |
| **DiSVA 426** | 0.0 ± 0.0 | ^c^ | 0.0 ± 0.0 | ^b^ | 50.0 ± 0.0 | ^b^ | 50.0 ± 0.0 | ^b^ | 2.2 ± 1.3 | ^i^ | 79.8 ± 0.1 | ^e^ | 41.9 ± 0.0 | ^f^ | 15.5 ± 0.61 | ^g^ | 30.7 ± 0.2 | ^k^ |
| **DiSVA 2** | 100.0 ± 0.0 | ^a^ | 0.0 ± 0.0 | ^b^ | 100.0 ± 0.0 | ^a^ | 50.0 ± 0.0 | ^b^ | 34.1 ± 1.4 | ^b^ | 82.6 ± 0.8 | ^cd^ | 45.6 ± 0.3 | ^e^ | 11.9 ± 1.81 | ^h^ | 99.0 ± 2.0 | ^a^ |
| **DiSVA 671** | 0.0 ± 0.0 | ^c^ | 0.0 ± 0.0 | ^b^ | 0.0 ± 0.0 | ^c^ | 50.0 ± 0.0 | ^b^ | 5.9 ± 0.0 | ^h^ | 73.1 ± 1.6 | ^gh^ | 35.7 ± 0.1 | ^h^ | 4.1 ± 1.62 | ^i^ | 43.7 ± 0.1 | ^h^ |
| **BOTECTOR** | 50.0 ± 0.0 | ^b^ | 0.0 ± 0.0 | ^b^ | 50.0 ± 0.0 | ^b^ | 50.0 ± 0.0 | ^b^ | 38.5 ± 0.6 | ^a^ | 73.5 ± 0.6 | ^gh^ | 0.0 ± 0.0 | ^o^ | 29.0 ± 0.0 | ^d^ | 31.2 ± 1.3 | ^k^ |
| **CODEX** | 0.0 ± 0.0 | ^c^ | 0.0 ± 0.0 | ^b^ | 0.0 ± 0.0 | ^c^ | 0.0 ± 0.0 | ^c^ | 0.0 ± 0.0 | ^j^ | 83.6 ± 0.2 | ^c^ | 29.0 ± 0.0 | ^j^ | 0.0 ± 0.0 | ^j^ | 27.0 ± 1.8 | ^l^ |

**Supplementary table s1.** Antimicrobial and antimold activity mediated by volatile organic compounds (VOCs) produced by the strains against bacterial (*E. coli. S. aureus. S. enteritidis. L. monocytogenes*) and mold (*A. carbonarius. B. cinerea. P. expansum. P. digitatum. Cladosporium*) targets. Data are means ± standard deviations. Values displaying different superscript letters within each column are significantly different according to Duncan’s test (p < 0.05).
